# The Mammalian Ecdysoneless Protein Interacts with RNA Helicase DDX39A To Regulate Nuclear mRNA Export

**DOI:** 10.1128/MCB.00103-21

**Published:** 2021-06-23

**Authors:** Irfana Saleem, Sameer Mirza, Aniruddha Sarkar, Mohsin Raza, Bhopal Mohapatra, Insha Mushtaq, Jun Hyun Kim, Nitish K. Mishra, Mansour A. Alsaleem, Emad A. Rakha, Fang Qiu, Chittibabu Guda, Hamid Band, Vimla Band

**Affiliations:** a Department of Genetics, Cell Biology and Anatomy, University of Nebraska Medical Center, Omaha, Nebraska, USA; b Department of Biochemistry and Molecular Biology, University of Nebraska Medical Center, Omaha, Nebraska, USA; c Department of Pathology and Microbiology, University of Nebraska Medical Center, Omaha, Nebraska, USA; d Department of Biostatistics, University of Nebraska Medical Center, Omaha, Nebraska, USA; e Eppley Institute for Research in Cancer and Allied Diseases, University of Nebraska Medical Center Omaha, Nebraska, USA; f Fred & Pamela Buffett Cancer Center, University of Nebraska Medical Center, Omaha, Nebraska, USA; g Department of Pathology, School of Medicine, University of Nottingham, Nottingham, United Kingdom

**Keywords:** ECD, ecdysoneless, hSGT1, ErbB2, RNA processing, RNA export, RNA splicing, RNA helicase, oncogenesis

## Abstract

The mammalian orthologue of ecdysoneless (ECD) protein is required for embryogenesis, cell cycle progression, and mitigation of endoplasmic reticulum stress. Here, we identified key components of the mRNA export complexes as binding partners of ECD and characterized the functional interaction of ECD with key mRNA export-related DEAD BOX protein helicase DDX39A. We find that ECD is involved in RNA export through its interaction with DDX39A. ECD knockdown (KD) blocks mRNA export from the nucleus to the cytoplasm, which is rescued by expression of full-length ECD but not an ECD mutant that is defective in interaction with DDX39A. We have previously shown that ECD protein is overexpressed in ErbB2^+^ breast cancers (BC). In this study, we extended the analyses to two publicly available BC mRNA The Cancer Genome Atlas (TCGA) and Molecular Taxonomy of Breast Cancer International Consortium (METABRIC) data sets. In both data sets, ECD mRNA overexpression correlated with short patient survival, specifically ErbB2^+^ BC. In the METABRIC data set, ECD overexpression also correlated with poor patient survival in triple-negative breast cancer (TNBC). Furthermore, ECD KD in ErbB2^+^ BC cells led to a decrease in ErbB2 mRNA level due to a block in its nuclear export and was associated with impairment of oncogenic traits. These findings provide novel mechanistic insight into the physiological and pathological functions of ECD.

## INTRODUCTION

Eukaryotic gene function requires precise coordination of transcription with mRNA processing, including splicing, capping, and polyadenylation, and efficient export through the nuclear pore complex to transfer the mRNAs to the cytoplasm where the machinery for translation resides (1–5). The kinetics and efficiency of the export of a mature mRNA from the nucleus to the cytoplasm require elaborate export machinery (4, 6–8). Key components of this machinery include the nuclear pore complex proteins (NPCs), RNA-binding/non-RNA-binding adaptor proteins, chaperones, and cochaperones inside the nucleus, which help the mRNAs to transverse the nuclear envelope and reach the cytoplasmic compartment (1, 2, 8–10). Two major mRNA export pathways are the nuclear RNA export factor 1 (NXF1)–NTF2-related export protein 1 (NXT1)-mediated bulk mRNA export pathway and the CRM1/XPO1-mediated selective mRNA export pathway (11–22). Both export pathways function in association with multiple adaptor proteins that associate with the mRNAs to form the mature messenger ribonucleoproteins (mRNPs) and help dock the mRNAs to the NPC channels for efficient export (4, 6, 18, 23, 24). The CRM1-mediated mRNA export pathway is more selective and facilitates the export of ribosomal RNAs, viral RNAs, U-snRNA, and other mRNAs using known as well as undefined adaptor proteins (1–8, 10, 17, 25–27).

Given the fundamental role of the mRNA export pathway in cell physiology and the importance of regulated mRNA transport under various physiological conditions and during pathological situations (2, 28, 29), the identification of novel components of the mRNA export machinery is of great interest. Here, we describe the mammalian ecdysoneless (ECD) protein as a novel structural and functional component of the mRNA export machinery.

The mammalian ECD is the highly conserved orthologue of *Drosophila* ecdysoneless (Ecd) whose mutations lead to developmental arrest due to loss of the metamorphosis-associated ecdysone hormone secretion during early development (50). *Drosophila* Ecd also functions cell autonomously in embryonic cell survival and was previously found to interact with the spliceosome factor pre-mRNA processing 8 (Prp8; orthologue of the mammalian PRPF8) (50), and loss of Prp8 or *Ecd* led to defective splicing of the ecdysone biosynthetic enzyme CYP307A2/spookier (spok) pre-mRNA, providing a basis for the metamorphosis defects in *ecd* mutant flies (30). Notably, human ECD could compensate for loss of *Drosophila Ecd* for this function.

We have previously shown that germ line deletion of *Ecd* in mice leads to early embryonic lethality and that recombinase-mediated deletion of *Ecd* in mouse embryonic fibroblasts (MEFs) from *Ecd^flox/flox^* (*Ecd^fl/fl^*) mice or the short hairpin RNA (shRNA)-mediated knockdown of *ECD* in human mammary epithelial cells leads to G_1_ cell cycle arrest, indicating an essential role of mammalian ECD in cell cycle progression (31). Recently, using depletion and overexpression approaches, we uncovered a role of ECD in mitigating endoplasmic reticulum stress through ECD-dependent attenuation of the PRKR-like endoplasmic reticulum kinase (PERK) branch of the unfolded protein response (32). We and others have shown that ECD interacts with the R2TP cochaperone complex (consisting of RUVBL1, RUVBL2, RPAP3, and PIH1D1 proteins), which functions in the assembly and remodeling of multimeric protein-RNA complexes, such as the U5 small nuclear ribonucleoprotein (snRNP) complex (33–37). Notably, mammalian PRPF8 also interacts with ECD (37), and another study found ECD, PRPF8, and R2TP subunits in a single complex (35). These studies have begun to point to potential roles of ECD in RNA biogenesis.

While the crystal structure of ECD is not known, our previous analysis using circular dichroism measurements and sequence analysis software showed that the majority of ECD is composed of α-helices and that the C-terminal 100 or so amino acids are disordered in the absence of binding partners. Furthermore, small-angle X-ray scattering (SAXS) analysis showed that the first 400 residues are globular and the next 100 residues are in an extended cylindrical structure (33), suggesting ECD acts like a structural hub or scaffolding protein in its associations with protein partners.

The importance of understanding the mechanism of how ECD functions is further highlighted by studies by us and others that have demonstrated ECD overexpression in several human cancers, such as those of pancreas, breast, and gastric tumors (38–41). We have shown that ECD overexpression in breast cancer patients correlates with poor prognosis and shorter survival, especially in the ErbB2^+^ breast cancer subtype (39). These studies support the likelihood of ECD’s role in promoting oncogenesis, a possibility supported by the ability of overexpressed ECD to cooperate with mutant H-Ras to oncogenically transform nontumorigenic immortal human mammary epithelial cells (42).

Here, we identify components of the mRNA export machinery as interacting partners of ECD and show that ECD regulates mRNA export. We previously showed that ECD protein is overexpressed in ErbB2^+^ breast cancers (BC). Furthermore, using The Cancer Genome Atlas (TCGA) and Molecular Taxonomy of Breast Cancer International Consortium (METABRIC) data sets, we show ECD mRNA overexpression correlates with short patient survival, specifically in ErbB2^+^ as well as triple-negative breast cancer (TNBC) patients, and serves as an independent prognostic marker. In ErbB2^+^ BC cells, ECD regulates ErbB2 mRNA export and stability and is required for ErbB2^+^ breast cancer cell proliferation, anchorage-independent growth, migration, and invasion. Thus, our findings provide new mechanistic insight into the physiological role of ECD and a potential basis for how overexpressed ECD may promote oncogenesis.

## RESULTS

### ECD interacts with components of the mRNA export machinery.

To gain insights into mechanisms by which mammalian ECD functions, we used two complementary approaches to identify ECD-associated cellular proteins. In one approach, we used a recombinant ECD protein with an N-terminal glutathione transferase (GST) and a C-terminal FLAG tag in an *in vitro* tandem affinity purification approach to identify cellular proteins that specifically bind to ECD; in the second approach, we expressed a FLAG-tagged ECD in HEK-293T cells and carried out affinity purification of ECD-associated proteins. In both approaches, mass spectrometry was used to identify ECD-associated proteins. A number of known as well as novel interacting proteins involved in various cellular processes were identified, including proteins involved in transcription, RNA processing, translation, ATP transport, cytoskeleton, metabolism, kinase pathways, and vesicle-mediated transport (see Table S1A in the supplemental material). Significantly, ECD-associated protein complexes from *in vitro* and *in vivo* approaches (listed above) include multiple proteins involved in mRNA export, such as U5 small nuclear ribonucleoprotein component (EFTUD2), BRR2, DDX39A/B, and LRPPRC as well as PRPF8 (Table S1A and B, highlighted in bold font), which was previously implicated in ECD’s role in mRNA splicing in *Drosophila* (30) and whose association with ECD in mammalian cells we reported previously (37).

**FIG 1:**
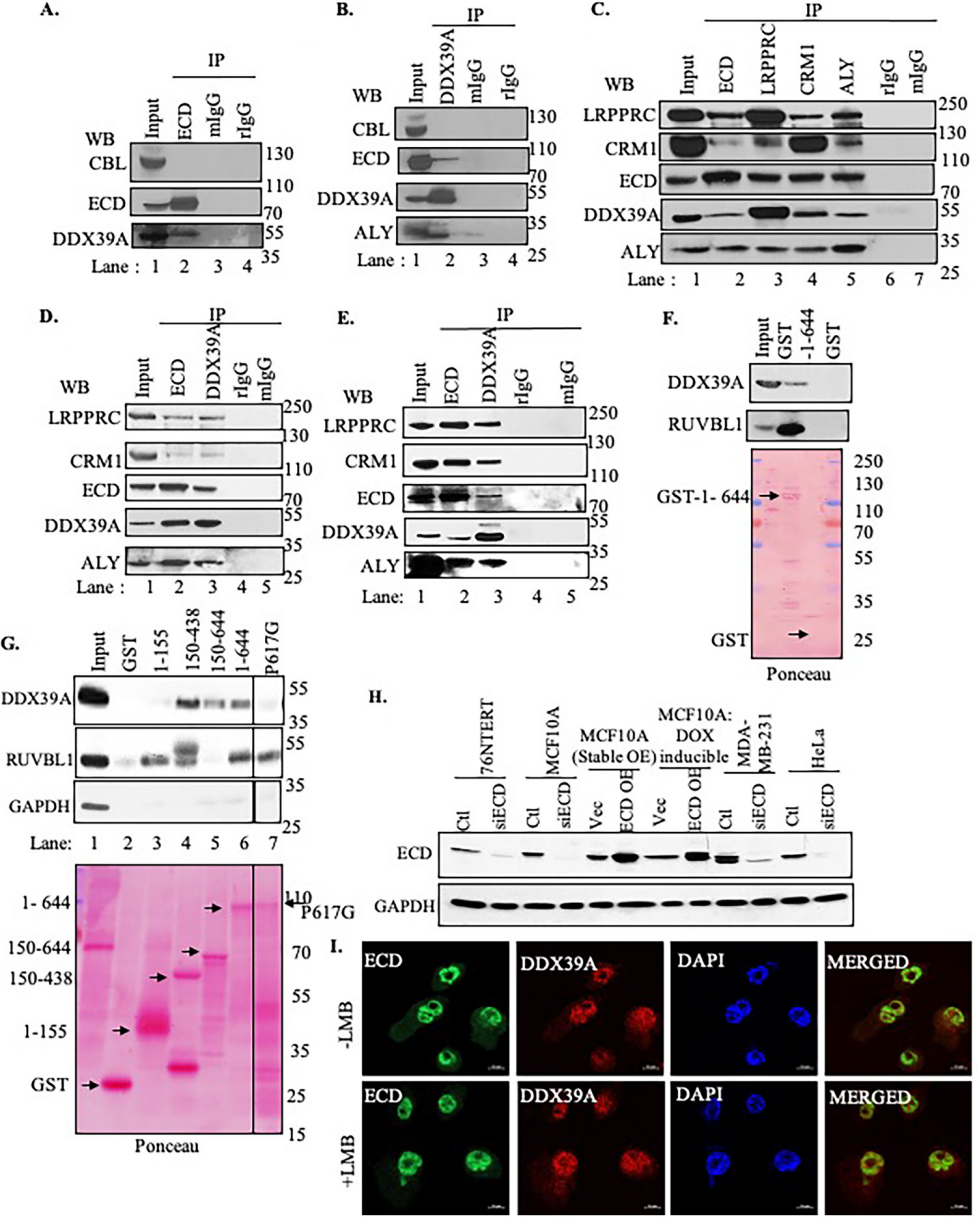
ECD interacts with DDX39A and other components of the mRNA export machinery. (A to E) HEK-293T (A to D) or MCF10A (E) cell lysates were immunoprecipitated (IP) with the antibodies indicated at the top, followed by Western blotting (WB) with the antibodies shown on the left. ALY and CRM1 were used as positive controls. CBL, mouse IgG (mIgG), and rabbit IgG (rIgG) were used as negative controls; 100-μg aliquots of lysate protein were used in the input lane. (F) Twenty micrograms of GST (negative control) or GST fusion with full-length ECD (1 to 644 aa) was incubated with 1 mg of protein lysate from HEK-293T cells transfected with FLAG-tagged RUVBL1 or FLAG-tagged DDX39A, and the GST pulldown proteins were analyzed by Western blotting with the indicated antibodies. The membrane was stained with Ponceau S to visualize the GST fusion proteins to assess comparable fusion protein use for pulldowns (indicated by arrows). (G) GST or GST fusion with full-length ECD (1 to 644) or indicated ECD mutants were incubated with protein lysate of HEK-293T cells transiently transfected with FLAG-tagged-DDX39A and FLAG-tagged RUVBL1, and the bound proteins were analyzed by Western blotting with the indicated antibodies. The membrane was stained with Ponceau S to visualize the GST fusion proteins (indicated by arrows). The experiment shown is a representative of at least three repeats with comparable results. (H) To validate ECD commercial antibody, Western blotting was performed in various cell lysates from control and ECD siRNA-treated MCF10A, 76NTERT, MDA-MB-231 and HeLa cells and ECD-overexpressing MCF10A stable as well as DOX-inducible (as indicated); glyceraldehyde-3-phosphate dehydrogenase (GAPDH) was used as loading control. (I) ECD colocalizes with DDX39A in the nucleus. MCF10A cells untreated or treated with 20 ng/μl of leptomycin B (LMB) for 4 h and fixed in 4% paraformaldehyde (PFA) were subjected to immunofluorescence staining using anti-ECD rabbit polyclonal and anti-DDX39 mouse monoclonal antibodies followed by imaging using 63× Zeiss confocal microscope (scale bars, 10 μm).

In view of a lack of previous linkage between ECD and mRNA export, studies here focused on further characterization of the interaction of ECD with DDX39A RNA helicase, an ECD-associated protein previously established as a critical player in mRNA export (43–47). We have already shown that ECD localizes to both nucleus and cytoplasm and harbors a very strong CRM1-mediated nuclear export signal and that, with leptomycin B treatment, exogenous ECD is retained in the nucleus (48). To confirm the interactions identified by mass spectrometry, we first carried out immunoprecipitations (IPs) of endogenous proteins followed by Western blotting (WB) for the potential interacting partners. IP from lysates of HEK-293T cells with antibodies against ECD (Fig. 1A) or DDX39A (Fig. 1B) versus rabbit (rIgG) or mouse (mIgG) IgG (as negative controls) followed by WB for ECD, DDX39A, or Casitas B-lineage lymphoma (CBL; an expected noninteracting control) showed that ECD coimmunoprecipitated with DDX39A and ALY, the latter a known interacting partner of DDX39A (49) that served as a positive control. Next, we expanded our analyses by carrying ECD, LRPPRC, CRM1, ALY, or IgG IPs from lysates of HEK-293T (Fig. 1C and D) or MCF10A (a spontaneously immortalized mammary epithelial cell line) (Fig. 1E) cell lysates and subjected these to WB with antibodies against CRM1, LRPPRC, ALY, DDX39A, or ECD (Fig. 1C). Conversely, we performed IP with anti-ECD or anti-DDX39A antibodies followed by WB with antibodies against the various mRNA export-associated proteins (Fig. 1D and E). These analyses demonstrated the association of endogenous ECD with DDX39A and with its associated protein complex (ALY, LRPPRC, and CRM1) in both cell lines (Fig. 1A to E). Co-IP of CRM1 served as a positive control for LRPPRC IP, as reported earlier (24), and co-IP of ALY served as a positive control for DDX39A IP.

In the second approach to confirm the interaction of ECD with DDX39A, we carried out pulldown experiments with GST fusion protein of ECD (amino acids 1 to 644) or GST alone (negative control) from lysates of HEK-293T cells transiently transfected to overexpress FLAG-DDX39A or FLAG-RUVBL1 (known binding partner of ECD) (37). WB of pulldowns confirmed the binding of ECD to DDX39A as well as to RUVBL1 (Fig. 1F). Next, to assess the regions of ECD that are required for binding to DDX39A, we used pulldowns with the GST fusion protein of full-length ECD (1 to 644) versus its various fragments (amino acids 1 to 155, 150 to 438, or 150 to 644) and ECD-P617G, which has single amino acid change (glycine in place of a proline conserved in multiple organisms and also known to cause molting defects in *Drosophila* upon mutation to other amino acid [50]). The deletion mutants ECD_150–438_ and ECD_150–644_ pulled down similarly to full-length ECD (FL-ECD; amino acids [aa] 1 to 644), while an N-terminal ECD fragment (ECD_1–155_) failed to bind to DDX39A (Fig. 1G), suggesting that the ECD region encompassing amino acids 150 to 438 is required for interaction with DDX39A. Notably DDX39A binding was abolished in the ECD-P617G mutant (Fig. 1G and Fig. S1); however, the single-amino-acid-change ECD mutant retained the ability to associate with RUVBL1, arguing against gross misfolding of this protein. The inability of this mutant to bind DDX39A suggests a potential modulation of the ECD-DDX39A interaction by the C-terminal region harboring the mutation, but the mechanism of such modulation remains unclear.

In addition, we also performed immunofluorescence analysis to assess if ECD is colocalized in the nucleus with DDX39A. As reported previously, ECD shuttles between the nucleus and cytoplasm, and leptomycin B treatment blocks nuclear export of ECD (48, 51). As our monoclonal antibody (39, 51) recognizes the cytoplasmic pool of ECD, we used a commercially available antibody against ECD. We first validated the specificity of this antibody in WB (Fig. 1H), followed by assessing if ECD colocalizes with DDX39A. These experiments showed leptomycin B treatment of cells results in predominantly nuclear localization of ECD, which colocalized with DDX39A (Fig. 1I).

**FIG 2:**
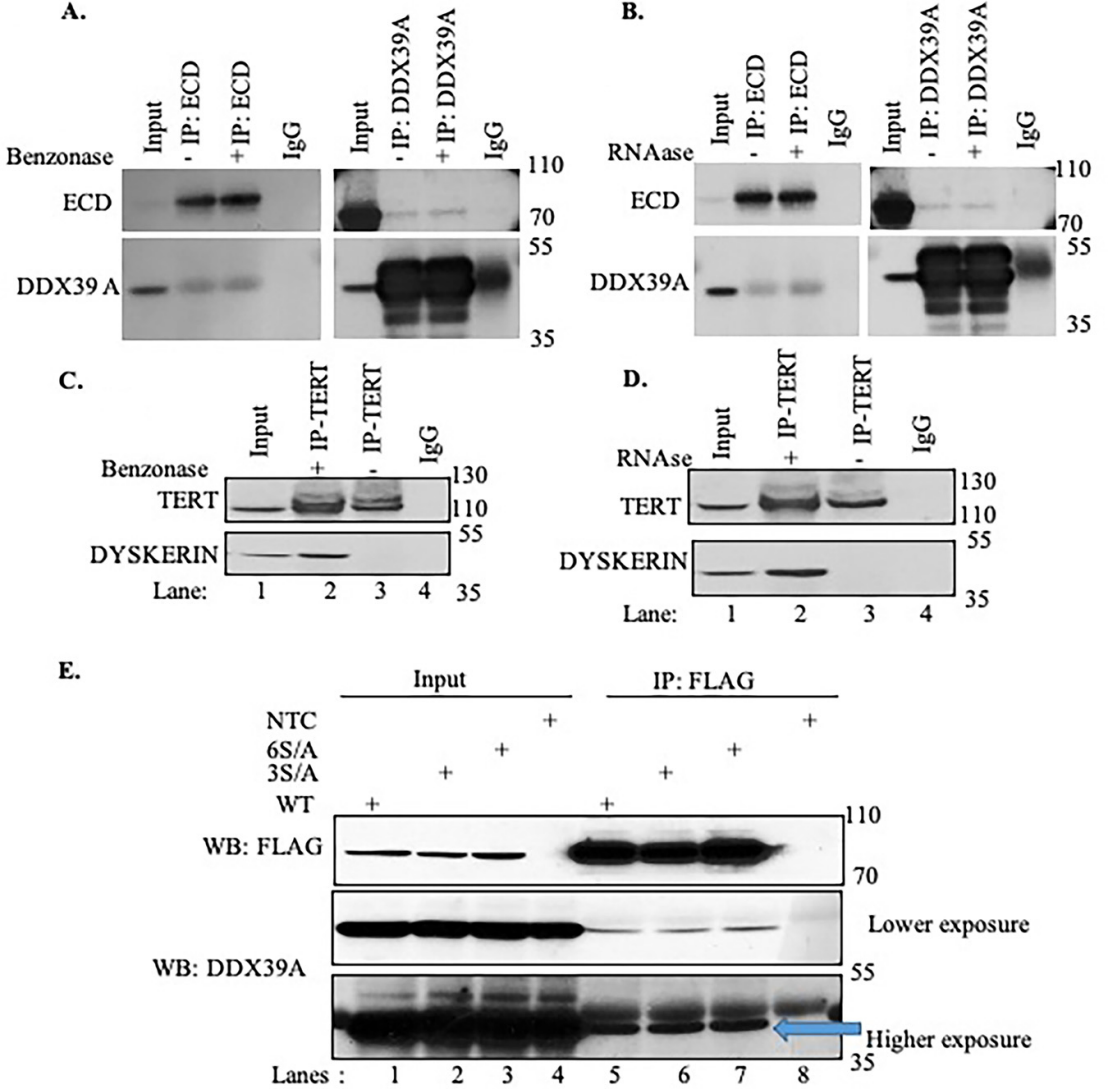
RNA-independent interaction of ECD with DDX39A. (A to D) MCF10A cell lysates treated with Benzonase/RNase were immunoprecipitated (IP) with the antibodies indicated at the top, followed by Western blotting (WB) with the antibodies shown on the left. TERT and dyskerin were used as positive controls. Mouse IgG (mIgG) and rabbit IgG (rIgG) were used as negative controls; 2% aliquots of lysate protein were used in the input lanes. (E) Phosphorylation-independent interaction of ECD with DDX39A. 293T cells were transfected with FLAG-tagged WT (wild-type) ECD or FLAG-tagged ECD phosphorylation site mutants 3S/A (503S/A, 505S/A, and 518S/A) and 6S/A (503S/A, 505S/A, 518S/A, 572S/A, 579S/A, and 584S/A) or untreated (nontransfected cells [NTC]). Cell lysates were immunoprecipitated (IP) with anti-FLAG antibody coupled to M2 FLAG-agarose beads (Sigma-Aldrich), followed by Western blotting (WB) with the antibodies shown on the left; 2% aliquots of lysate protein were used in the input lanes. Blue arrow indicates the DDX39A band at higher exposure.

As DDX39A is an RNA-binding protein, we further analyzed whether associations of DDX39A with ECD are RNA dependent by treating cell lysates with either RNase or Benzonase followed by IP/WB. We observed that ECD interaction with DDX39A is RNA independent (Fig. 2A to D). In these experiments, we used telomerase reverse transcriptase (TERT) and dyskerin association, a known RNA-dependent interaction, as our positive control (Fig. 2C and D). Furthermore, our previous studies have shown ECD is a phosphoprotein, and casein kinase 2 phosphorylates ECD at multiple sites (37). To assess if phosphorylation of ECD is required for its interaction with DDX39A, we used two phosphomutants of ECD and analyzed their association with DDX39A. As shown in Fig. 2E, phospho-deficient mutants associated with DDX39A similarly to wild-type ECD. Thus, ECD phosphorylation is not required for its association with DDX39A.

Taken together, our analyses established that ECD interacts with DDX39A and its associated nuclear export protein complex.

### ECD regulates nuclear mRNA export to the cytoplasm.

**FIG 3:**
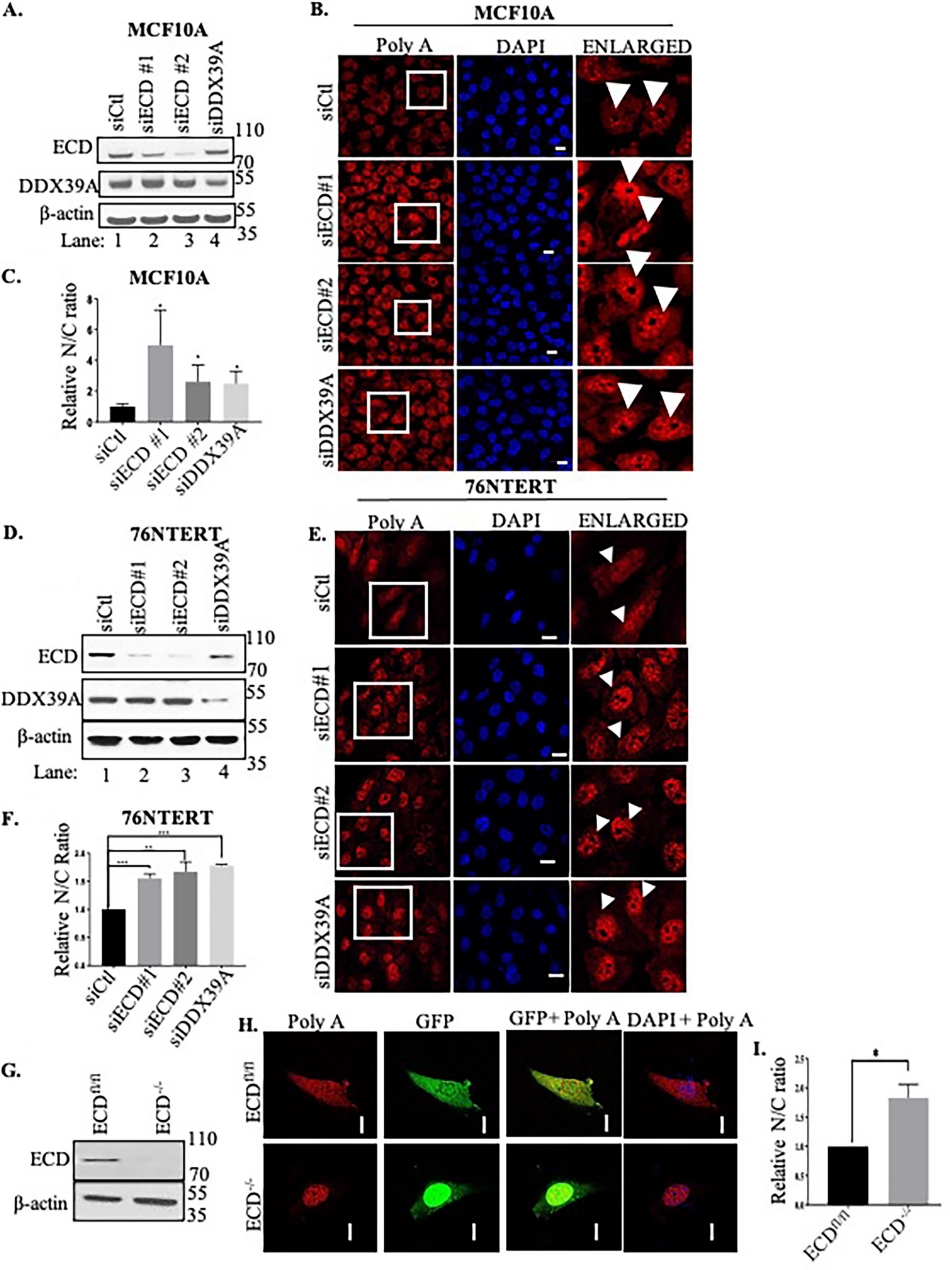
ECD KD decreases poly(A) mRNA export from the nucleus to the cytoplasm. (A to F) RNA FISH analysis in MCF10A (A to C) and 76NTERT (D to F) cells with control siRNA (Ctl; as a negative control), two independent siECDs (number 1 and number 2), or siDDX39A (as a positive control). (A and D) WB shows ECD or DDX39A KD. (B and E) Cells were fixed and hybridized with 12 μM oligo(dT) 22-nt Quasar 570 probe and then imaged using a LSM 710 Zeiss confocal microscope at ×63 magnification. Last column shows enlarged view of cells shown in the insets. (C and F) Quantification of nucleus-to-cytoplasmic signal ratio (N/C) was conducted using ImageJ software in at least 100 cells, and graphs were plotted by normalizing to the control cells to calculate the fold change. (G to I) *ECD^fl/fl^* cells were either treated with GFP (control) or Cre-GFP adenovirus to delete ECD. (G) WB shows Cre-mediated knockdown of ECD; β-actin was used as a loading control. (H) Cells were fixed and imaged as discussed above. (I) Quantifications of at least 25 cells using ImageJ to measure nucleus-to-cytoplasmic signal ratio (N/C) by normalizing to the control cells to calculate the fold change. Significance of the ratios was calculated using Student’s two-tailed *t* test; *, *P* ≤ 0.05, error bars represent standard errors from the mean (SEs). Means ± SEs were derived from three different experiments. Bars, 10 μm.

The interaction of ECD with DDX39A and associated proteins involved in mRNA export supported a potential functional role of ECD in regulating mRNA export from the nucleus to the cytoplasm. To address this possibility, we first examined the impact of ECD knockdown (KD) on mRNA export using fluorescence *in situ* hybridization (FISH) analysis of poly(A) mRNAs (26, 45, 52). Immortal mammary epithelial cell lines MCF10A and 76NTERT were transfected with control small interfering RNA (siRNA) or with two independent siRNAs against ECD or DDX39A (used as a positive control), and ECD or DDX39A KD was confirmed by Western blotting (Fig. 3A and D). FISH analysis of the relative abundance of nuclear versus cytoplasmic poly(A) mRNA revealed that DDX39A KD led to increased nuclear relative to cytoplasmic poly(A) mRNA signals in both cell lines, as expected (52). Notably, similar to that seen with DDX39A KD, ECD KD also led to a significant increase in nuclear poly(A) mRNA signals (Fig. 3B and E). Quantitation of the nuclear/cytoplasmic ratio of poly(A) mRNA FISH signals in 100 or more cells confirmed the nuclear accumulation of mRNA upon ECD KD (Fig. 3C and F).

To further validate the negative impact of ECD KD on poly(A) mRNA export, we utilized *Ecd^fl/fl^* mouse embryonic fibroblasts (MEFs) (31, 32) infected with adenoviruses encoding either green fluorescent protein (GFP) (control; expressed in cytoplasm and nucleus) or GFP-Cre recombinase (nucleus targeted) to induce the deletion of floxed *Ecd* (31). Western blotting confirmed the marked depletion of ECD expression in GFP-Cre versus that in the control GFP adenovirus-infected MEFs (Fig. 3G), and immunofluorescence analysis for GFP confirmed the expression of control GFP in both cytoplasm and nucleus and that of GFP-Cre in the nucleus (Fig. 3H). Notably, compared to a more uniform nuclear and cytoplasmic distribution in GFP-expressing control MEFs, a substantial nuclear accumulation of poly(A) mRNA was observed in GFP-Cre (Ecd-deleted) MEFs (Fig. 3H). Quantification of the nuclear/cytoplasmic ratio of poly(A) mRNA FISH signals from 25 cells revealed a significant impact of *Ecd* deletion (Fig. 3I). Collectively, these analyses support the conclusion that ECD is required for efficient export of poly(A) mRNA from the nucleus to the cytoplasm.

### ECD is involved in DDX39A-dependent mRNA export.

**FIG 4:**
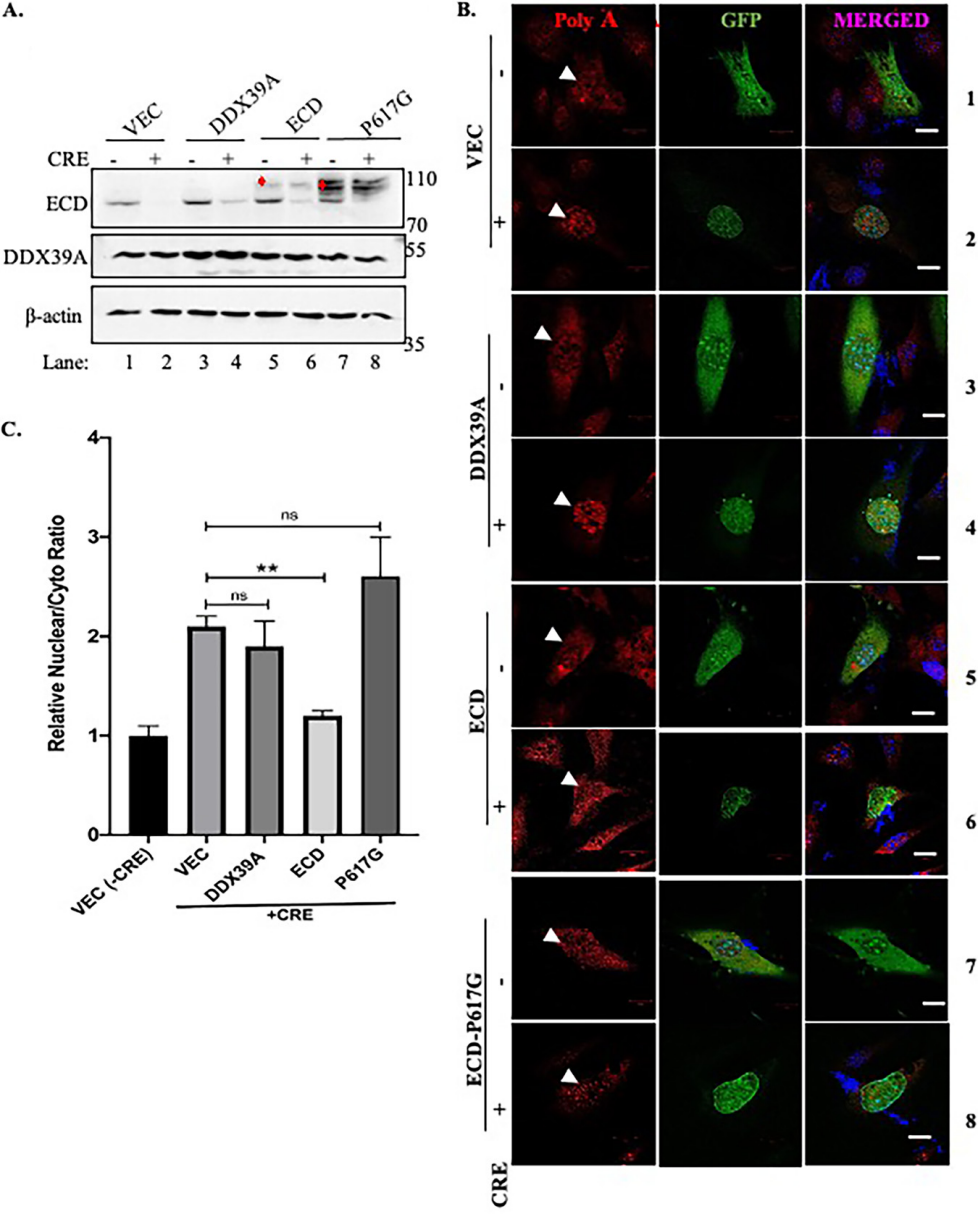
ECD interaction with DDX39A is required for mRNA export. RNA FISH analysis was performed on *Ecd^fl/fl^* MEFs overexpressing either vector, ECD, DDX39A, or ECD-P617G after treatment with GFP (control) or Cre-GFP adenovirus to delete ECD. (A) WB shows Cre-mediated knockdown of ECD. Exogenous ECD indicated by red marks. β-Actin was used as a loading control. (B) Cells were fixed and hybridized with 12 μM oligo(dT) 22-nt Quasar 570 probe and then imaged at ×63 magnification. Bars, 10 μm. (C) Quantifications of nucleus-to-cytoplasmic signal ratios were conducted using ImageJ software in at least 75 cells, and graphs were plotted by normalizing to the control cells. Significance of the ratios was calculated using Student’s two-tailed *t* test between groups as indicated, error bars represent standard errors from the means. *, *P* ≤ 0.05; **, *P* ≤ 0.01; ***, *P* ≤ 0.001.

Given our findings that ECD interacts with DDX39A and is required for mRNA export, we wished to examine if the ECD-DDX39A interaction is functionally important for the mRNA export function of ECD. First, we assessed if ECD KD had any impact on the levels DDX39A and other mRNA export-related proteins. ECD KD did not affect the levels or localization of DDX39A or its associated partners (data not shown). Next, we compared the ability of exogenous ECD or its DDX39A-noninteracting mutant P617G to rescue the defective mRNA export induced upon Cre-mediated deletion of *Ecd* in *Ecd^fl/fl^* MEFs. GFP expression was used to identify cells infected with control GFP or GFP-Cre adenovirus (green cells). Western blotting confirmed the expression of exogenous FLAG-tagged ECD, ECD-P617G mutant, or DDX39A versus that of the vector-alone transduced cells (Fig. 4A). RNA FISH analysis for poly(A) mRNA showed the expected nuclear accumulation of poly(A) mRNA in GFP-Cre-infected cells transduced with vector alone (Fig. 4B, row 2 versus row 1). Notably, MEFs with *Ecd* deletion that were transduced with human ECD showed nuclear/cytoplasmic poly(A) mRNA signals comparable to those without *Ecd* deletion (seen as red dots in the cytoplasm) (Fig. 4B, compare rows 5 and 6), indicating rescue of the mRNA export block. In contrast, *Ecd*-deleted MEFs transduced with ECD-P617G mutant did not show a rescue of the poly(A) mRNA export block induced by *Ecd* deletion (Fig. 4B, compare rows 7 and 8). Notably, expression of exogenous DDX39A expression did not rescue the mRNA export block induced by *Ecd* deletion (Fig. 4B, compare rows 3 and 4), indicating that ECD may function downstream of DDX39A in mRNA export. Quantification of nuclear/cytoplasmic mRNA signals normalized to −Cre and vector alone confirmed rescue of *Ecd* deletion-induced mRNA export block by ECD but not by ECD-P617G mutant or DDX39A (Fig. 4C). Taken together, these results demonstrate that interaction of ECD with DDX39A is required for its role in mRNA export and that ECD plays a distinct role that cannot be fulfilled by DDX39A itself.

Given our previous findings that ECD protein is overexpressed in cancers (38, 39), including breast cancers (BC), we wished to analyze if overexpression of ECD protein is due to increased mRNA levels and if mRNA export function of ECD relates to its oncogenic function in breast cancer cells.

### ECD mRNA is overexpressed in breast cancer patients, and its mRNA overexpression serves as an independent prognostic marker that predicts survival in breast cancer patients.

**FIG 5:**
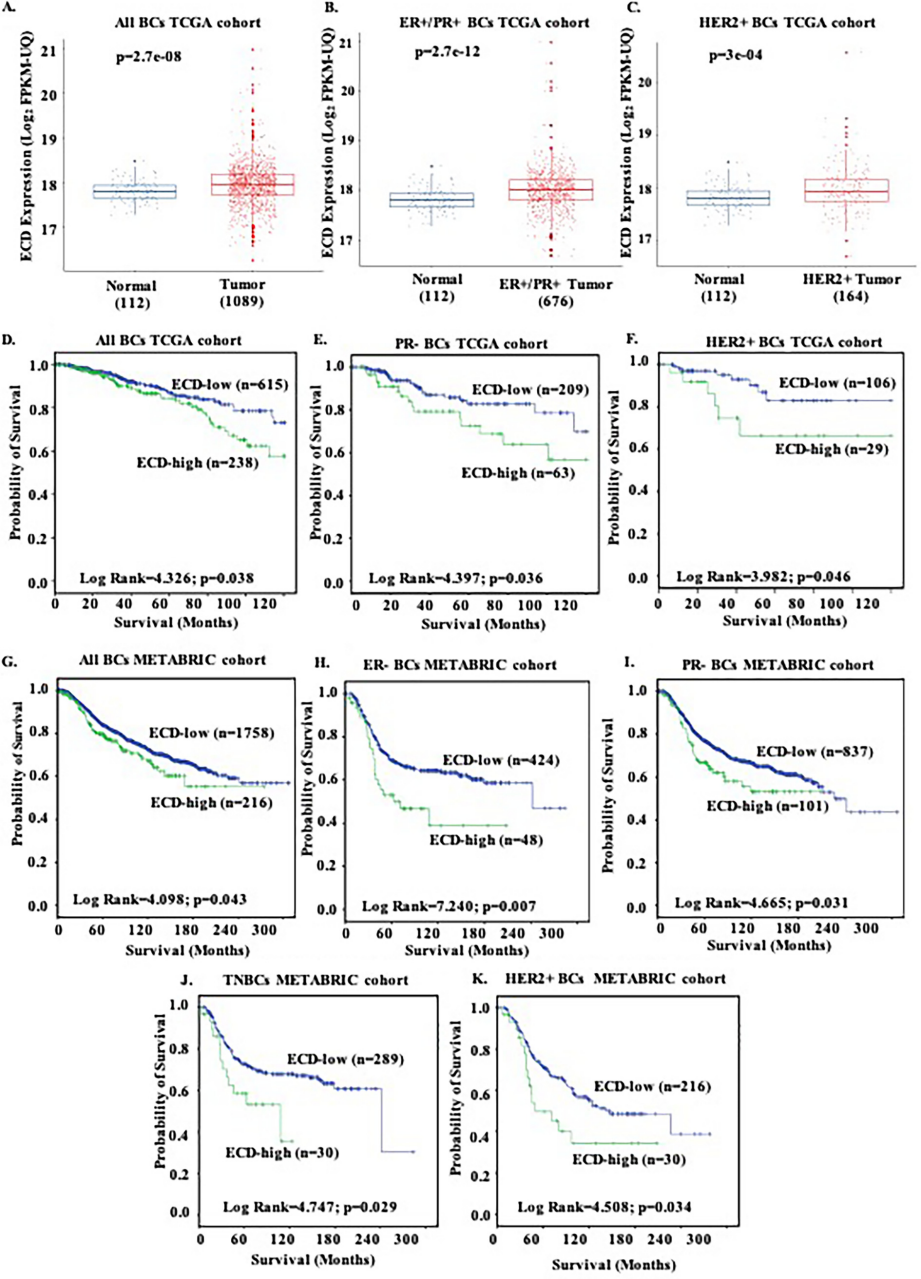
ECD mRNA analyses in breast cancer The Cancer Genome Atlas (TCGA) and the Molecular Taxonomy of Breast Cancer International Consortium (METABRIC) data sets reveal ECD mRNA overexpression correlates with poor patient survival. (A) TCGA breast cancer data set comprising a total of 1,089 breast cancer cases, including 676 cases of ER^+^/PR^+^, 164 cases of HER2^+^, and 112 adjacent normal tissue samples, was analyzed for ECD mRNA expression. RNA-seq expression level read counts were normalized using the upper quartile fragments per kilobase to transcript per million mapped reads (FPKM-UQ) calculation, and ECD expression in box plots is represented as log base 2 compared to that in adjacent normal tissue. In TCGA cohorts, ECD mRNA expression is significantly higher in all breast cancer tissues than in adjacent normal breast tissues (*P* = 2.7e−08) (A), in ER^+^/PR^+^ breast cancer samples (*P* = 2.7e−12) (B), and in HER2^+^ breast cancer cases (*P* = 3e−04) (C). (D) The 10-year overall survival of 854 cases was significantly worse in ECD-high (238) than in ECD-low (616) mRNA-expressing patients. High ECD mRNA expression in PR^−^ subgroup (E) and HER2^+^ (F) subgroup of TCGA data set correlates with poor survival. (G) METABRIC cohort: Kaplan-Meier plot for survival curve of all the breast cancer patients shows poor prognosis with high ECD mRNA, including in the ER^−^ subgroup (H), PR^−^ subgroup (I), triple-negative breast cancer (TNBC) subgroup (J), and HER2^+^ subgroup (K). Cox regression analysis results are shown with the Kaplan-Meier plots.

Analyses of two publicly available data sets, METABRIC (expression based on microarray) and TCGA (expression based on transcriptome sequencing [RNA-seq]), showed that ECD is overexpressed in BC tissues compared to that in adjacent normal breast tissue samples, as shown in the TCGA data set (Fig. 5A). When molecular subtypes were considered, ECD overexpression was seen in the estrogen receptor-positive (ER^+^)/progesterone receptor-positive (PR^+^) (luminal class) and HER2^+^ BC subtypes (Fig. 5B and C). Survival analysis showed that ECD overexpression significantly correlates with poor BC patient survival in the whole cohorts as well as in the hormone receptor-negative, TNBC, and HER2^+^ subgroups (Fig. 5D to K), supporting and confirming our previous immunohistochemistry (IHC) analysis. In addition, higher ECD expression was associated with variables characteristic of poor prognosis, including lymphovascular invasion (LVI) (χ^2^ = 8.150, *P* = 0.004) and higher Nottingham prognostic index (NPI) scores, and with the luminal B subtype and the integrative clusters 5 and 6 (see Table S2A). To further evaluate the prognostic importance of *ECD* mRNA expression, we performed multivariate analyses with a model incorporating clinical features (age, tumor size, tumor grade, ER status, PR status, and HER2 status) in both TCGA and METABRIC cohorts, and *ECD* overexpression was shown to be an independent significant predictor of poor outcome (see Table S2B).

To examine whether there is a concordance between ECD mRNA and its protein expression, we assessed the correlation of ECD protein and mRNA expression, which shows a statistically significant positive linear association between ECD protein and mRNA expressions (*r* = 0.21, *P* = 0.011).

Taken together, these analyses in two independent BC data sets affirm that ECD mRNA overexpression is associated with adverse prognostic features and shorter survival of patients.

### ECD regulates ErbB2 mRNA export and stability.

**FIG 6:**
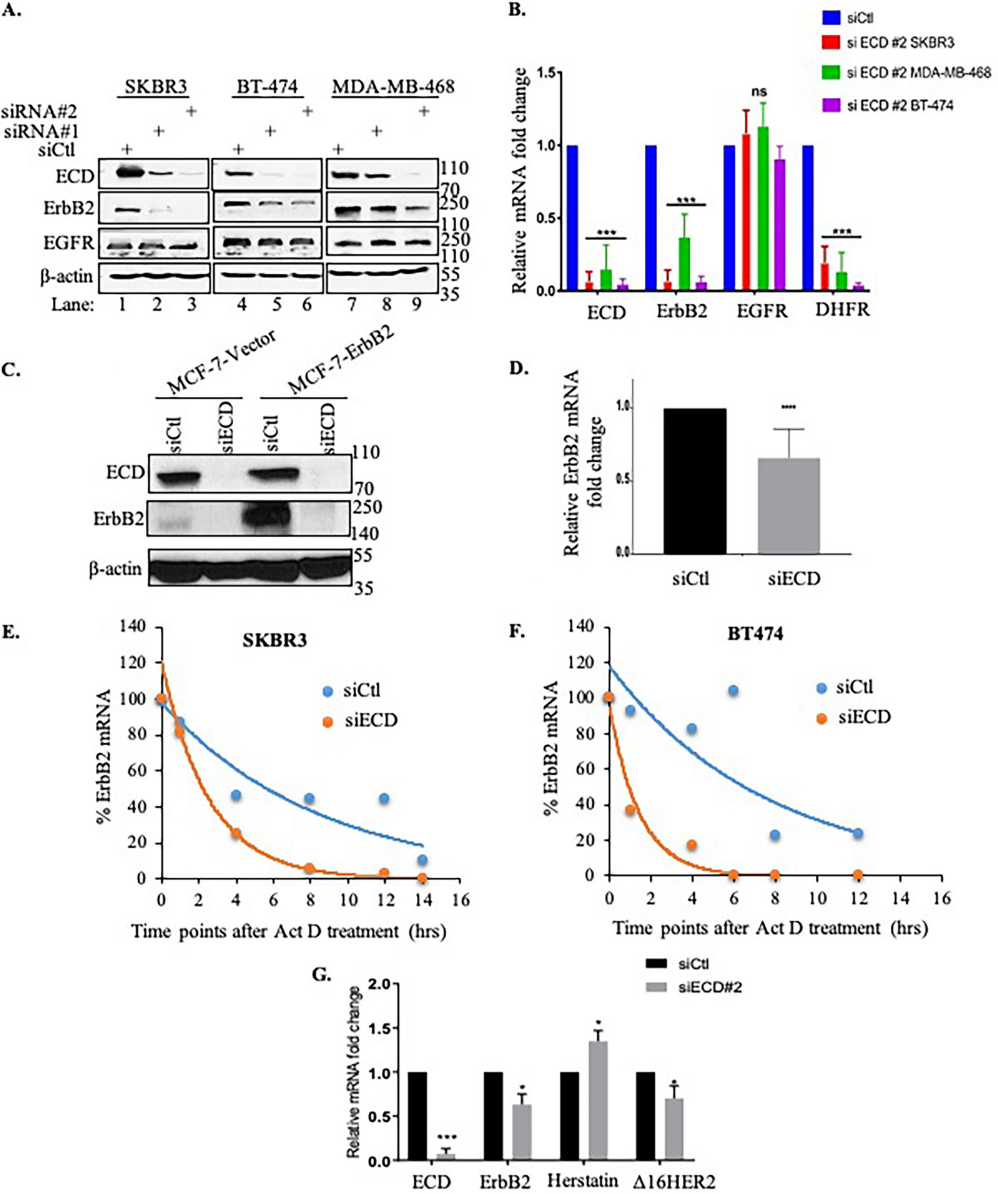
ECD KD decreases ErbB2 protein and mRNA expression in breast cancer cells. ECD knockdown was performed using specific siRNA against ECD in BT-474, SKBR3 (ErbB2-overexpressing), and MDA-MB-468 (EGFR-overexpressing) breast cancer cell lines. Protein and total mRNA were isolated after 48 h of transfection. (A) Lysates were harvested and immunoblotted with the indicated antibodies. β-Actin was used as a loading control. (B) Control mRNA levels of ECD, ErbB2, or EGFR were detected by qRT-PCR; DHFR was used as a positive control, and GAPDH served as an internal control. Fold change over GAPDH was calculated and plotted and normalized with control. (C) MCF-7-vector and MCF-7-ErbB2 expressing cells were treated with ECD siRNA or control siRNA, and lysates were collected and blotted with indicated antibodies. (D) RNA was isolated, cDNA was prepared, and qRT-PCR was performed using ErbB2-specific primers. Fold change with respect to control after normalizing with β-actin was calculated and plotted. ECD downregulation decreases ErbB2 mRNA stability. (E and F) The stability of mRNA was analyzed in SKBR3 and BT-474 breast cancer cell lines. After ECD downregulation by siRNA, cells were treated with actinomycin D (5 μg/ml) at zero time point, and then total RNA was isolated at various time points (0, 2, 4, 6, 8 and 12 h) after actinomycin D treatment. cDNA was made, and levels of ErbB2 were measured by qRT-PCR. GAPDH was used as normalization control. (G) qRT-PCR using specific primers of indicated genes from RNA samples of SKBR3 cells treated with control and ECD siRNA. ECD knockdown was confirmed. HER2 (full form) and its isoforms, herstatin and Δ16HER2, were analyzed using specific sets of primers. β-Actin was used as an internal control. Fold change with respect to control after normalizing with β-actin was calculated and plotted. Significance of the ratios was calculated using Student’s two-tailed *t* test. ns, not significant; *, *P* ≤ 0.05; **, *P* ≤ 0.01; ***, *P* ≤ 0.001. Error bars represent means ± standard deviations (SDs) from six replicates in a representative experiment. The experiment was repeated three times independently.

As the driver oncogene, ErbB2 mRNA biogenesis is pivotal for oncogenesis (53); thus, we wished to examine if ECD plays a role in ErbB2 mRNA export. First, we transfected ErbB2-overexpressing SKBR3 and BT-474 or epidermal growth factor receptor (EGFR)-overexpressing MDA-MB-468 BC cell lines with control or two independent ECD siRNAs and then examined the levels of ErbB2 and EGFR. ECD KD in both ErbB2-overexpressing cell lines (SKBR3 and BT-474), as well as in MDA-MB-468 (does not overexpress ErbB2), resulted in a reduction in ErbB2 protein levels (Fig. 6A). On the other hand, there was no change in EGFR protein levels in either the EGFR-overexpressing MDA-MB-468 cells or in other two cell lines that express lower basal EGFR levels (Fig. 6A). Notably, analyses of ErbB2 and EGFR mRNA levels using quantitative real-time PCR (qRT-PCR) showed that ECD KD resulted in a drastic reduction in ErbB2 mRNA levels (Fig. 6B). *DHFR*, an E2F target gene whose expression we have previously shown to be reduced in *Ecd* knockout MEFs (31), served as a positive control. Compared to a marked reduction in ErbB2 mRNA levels, no reduction was observed in EGFR mRNA levels upon ECD KD (Fig. 6B).

Next, we assessed if ECD is transcriptionally regulating ErbB2 by overexpressing exogenous ErbB2 under the cytomegalovirus (CMV) promoter in MCF-7 cells (express low levels of endogenous ErbB2). Vector- or ErbB2-expressing MCF-7 cells were treated with control siRNA or ECD siRNA, followed by analyses of RNA by qRT-PCR and protein by Western blotting. Significant decreases in ErbB2 mRNA and protein levels were observed upon ECD KD (Fig. 6C and D), excluding the transcriptional regulation of ErbB2 by ECD.

In addition, we examined if the decrease in ErbB2 mRNA levels upon ECD KD is due to decreased mRNA stability. For this purpose, two breast cancer cell lines, SKBR3 and BT-474, were treated with actinomycin D, a transcriptional blocker agent, after ECD KD. Total RNA was isolated at designated time points after actinomycin D treatment. qRT-PCR was performed to analyze ErbB2 mRNA levels. In ECD-downregulated cells, at zero time point, the ErbB2 mRNA levels were low compared to those in control cells. As time progressed, the mRNA in control cells was more stable. Notably, the ErbB2 mRNA half-life was approximately 7 to 8 h in scrambled siRNA-expressing SKBR3 and BT-474 cells, similar to previously published data (54, 55). However, ECD KD reduced the ErbB2 mRNA stability to approximately 2 to 3 h (Fig. 6E and F).

Previous studies showed ECD interacts with PRPF8 and has been identified as part of spliceosome complex; therefore, we assessed if ECD KD affects ErbB2 splice variants. We examined the expression of two well-documented splice variants, herstatin (generated by inclusion of intron 8) and Δ16HER2 (produced due to skipping of exon 16) (56–58). Notably, ECD KD resulted in alterations in the mRNA levels of both splice variants (Fig. 6G). Significantly, ECD KD led to an increase in the expression of herstatin, a tumor suppressor splice variant (58–60), but a reduction in the expression of the Δ16HER2 variant, a prooncogenic splice variant (57, 61, 62). Taken together, we demonstrate transcriptional regulation of ErbB2 and its splice variants by ECD.

**FIG 7:**
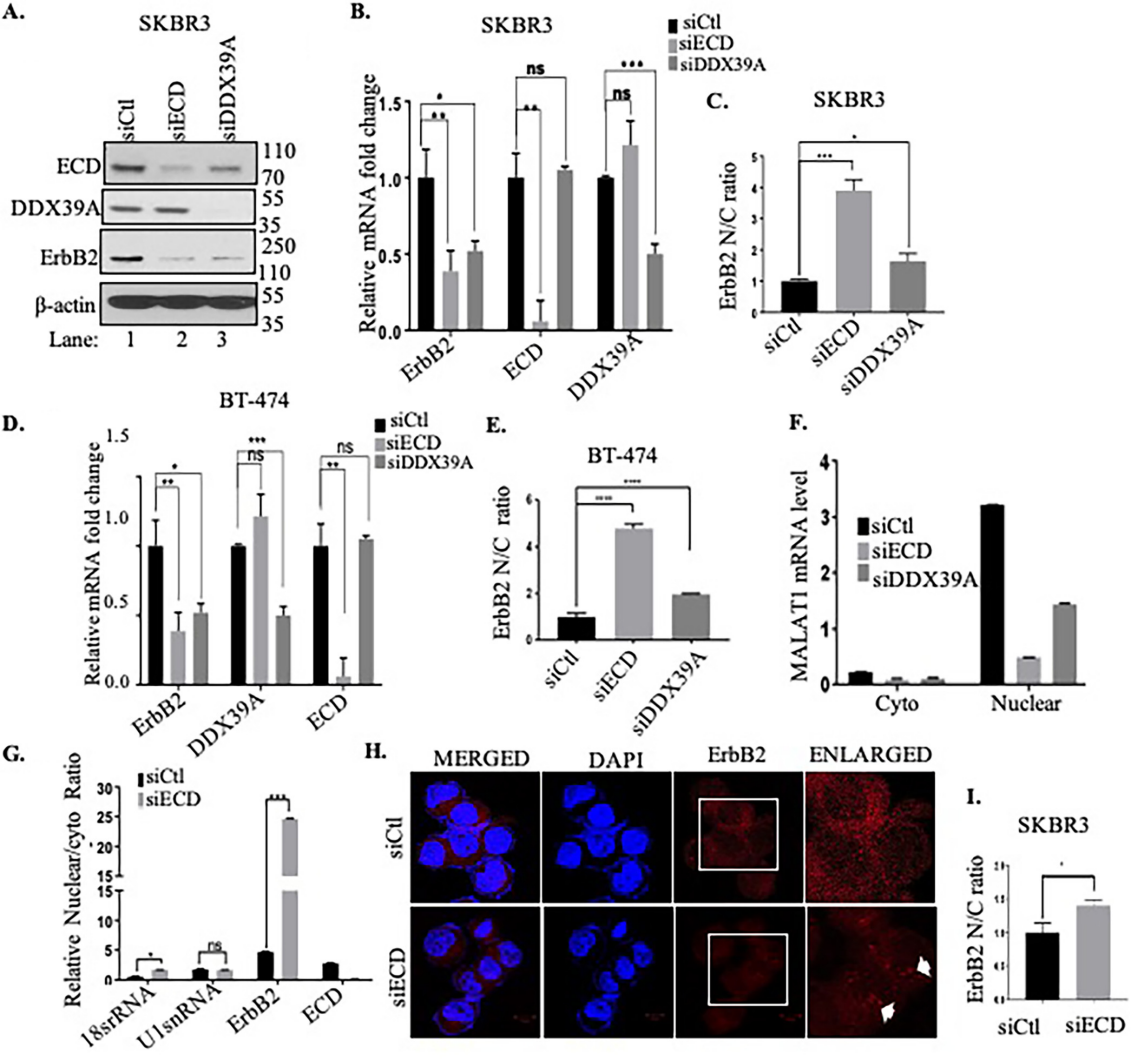
ECD KD increases nuclear ErbB2 mRNA accumulation. SKBR3 and BT-474 cells were transfected with control siRNA, ECD siRNA, and DDX39A siRNA. (A) Western blotting was performed with indicated antibodies; β-actin was used as a loading control, confirming knockdown of ECD and DDX39A in SKBR3. (B and D) qRT-PCR shows decrease in ErbB2 mRNA in ECD or DDX39A KD cells in comparison to control siRNA-treated cells. The graph plotted shows ECD, ErbB2 and DDX39A mRNA levels normalized to β-actin, and fold change was calculated by normalizing to control in SKBR3 (B) and in BT-474 (D) cells. SKBR3 cells were subjected to subcellular RNA fractionation after treating the cells with control siRNA, ECD siRNA, and DDX39A siRNA. (C and E) Subcellular RNA fractionation after control siRNA, ECD siRNA, or DDX39A siRNA treatment followed by qRT-PCR of ErbB2 mRNA showing nuclear/cytoplasmic ratio (N/C) in SKBR3 (C) and BT-474 (E) cells. (F) qRT-PCR of MALAT1 (a long noncoding RNA) used as control for purification of nuclear fraction. (G) qRT-PCR showing nucleus-to-cytoplasmic ratio of 18S rRNA, U1 snRNA, and ErbB2 and ECD mRNA in control siRNA- and ECD siRNA-treated cells; each cytoplasmic and nuclear fraction was normalized to corresponding total fraction. Significance was calculated using Student’s two-tailed *t* test. *, *P* ≤ 0.05; **, *P* ≤ 0.01; ***, *P* ≤ 0.00; ns, nonsignificant, error bars represent standard errors from the means. Means ± SEs were derived from three different experiments. ECD knockdown was performed using specific siRNA against ECD in SKBR3 cells. (H) RNA FISH analysis was carried out with 12 μM oligonucleotide anti-ErbB2 570 probe and then imaged at ×63 magnification; last column shows enlarged view of cells. White arrows show the accumulation of ErbB2 mRNA probe. (I) Quantifications of at least 25 cells using ImageJ showing nucleus-to-cytoplasmic ratio (N/C) by normalizing to the control cells to calculate the fold change. Significance of the ratios was calculated using Student’s two-tailed *t* test. *, *P* ≤ 0.05, error bars represent standard errors from the means. Means ± SEs were derived from three different experiments.

Next, we examined the effect of DDX39A KD on the levels of ErbB2 mRNA and protein. We observed similar decreases in ErbB2 protein (Fig. 7A) and mRNA (Fig. 7B) levels upon DDX39A KD as well as ECD KD. To assess if the decrease in ErbB2 mRNA levels upon ECD KD or DDX39A KD was due to a block in ErbB2 nuclear export, we quantified ErbB2 mRNA levels in nuclear versus cytoplasmic fractions of control versus ECD or DDX39A KD cells. The qRT-PCR analyses revealed a higher nuclear-to-cytoplasmic ratio of ErbB2 mRNA in ECD or DDX39A KD cells than in controls in SKBR3 (Fig. 7B and C) and in BT-474 (Fig. 7D and E) cells; supporting the conclusion that KD of ECD or DDX39A reduces the export of ErbB2 mRNA from the nucleus to the cytoplasm. The purity of nuclear and cytoplasmic fractions was confirmed by analyzing the long noncoding RNA MALAT1 (Fig. 7F) and 18s rRNA and U1 snRNA (Fig. 7G) (63).

Next, we performed ErbB2 mRNA FISH analysis using an ErbB2-specific RNA probe using SKBR3 cells. Consistent with the ECD requirement for ErbB2 mRNA export, the ECD KD cells showed an accumulation of ErbB2 mRNA in the nucleus (more red dots in the nucleus) (Fig. 7H). Quantification of the nuclear versus cytoplasmic ErbB2 mRNA signals showed a higher nuclear/cytoplasmic ratio of ErbB2 mRNA signals upon ECD KD (Fig. 7I).

Taken together, these experiments demonstrate that ECD is an essential protein required for ErbB2 mRNA processing from the nucleus to the cytoplasm.

### Requirement of ECD for oncogenic traits of ErbB2-overexpressing breast cancer cells.

**FIG 8:**
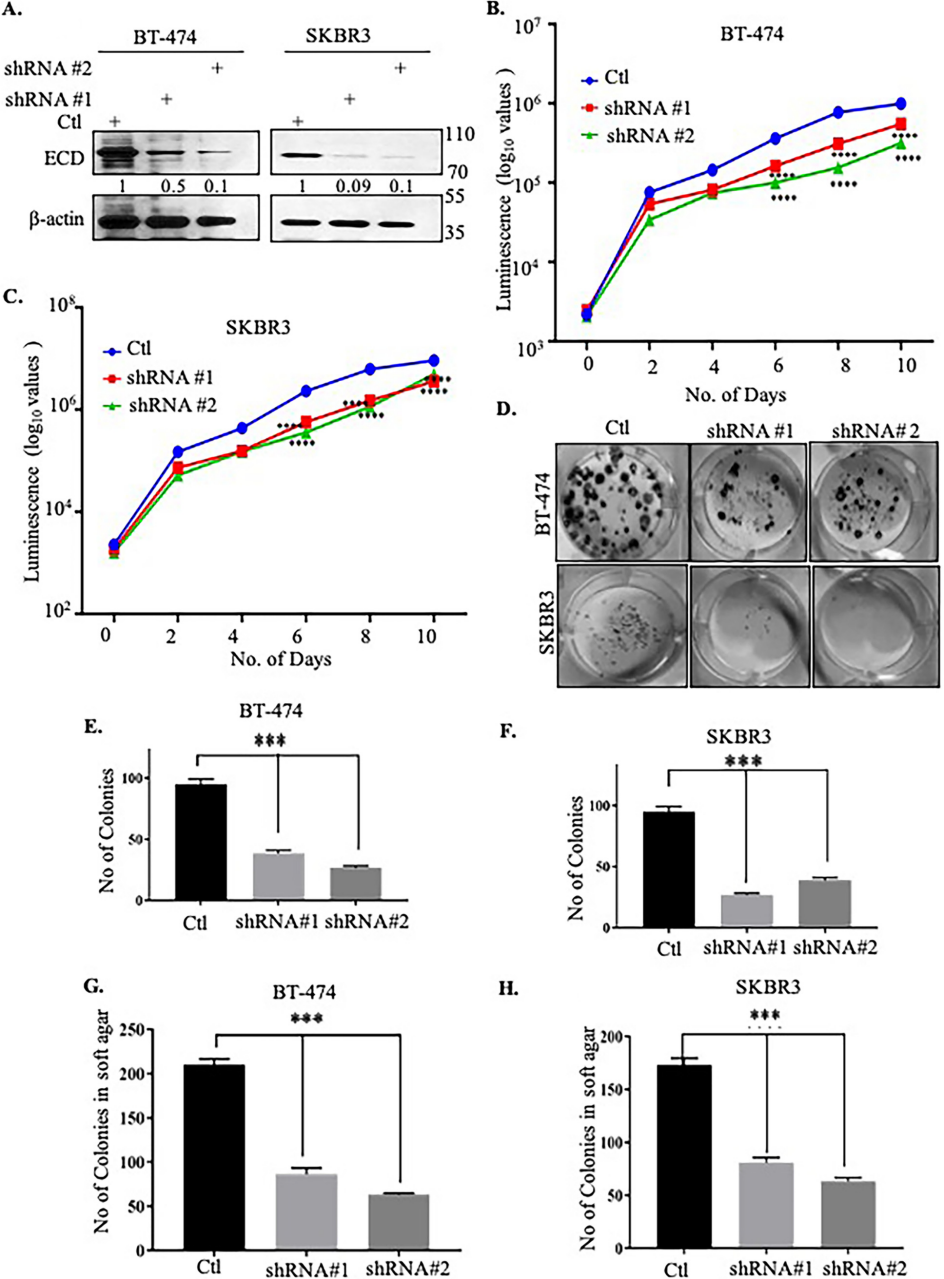
ECD knockdown decreases cell proliferation and anchorage independent of ErbB2-overexpressing breast cancer cells. (A) ECD was stably knockdown by two different shRNAs against ECD, and then KD was assessed by Western blotting. (B and C) Cell proliferation assays were performed using CellTiter-Glo luminescent cell viability assay; 2,000 cells per well were plated (as discussed in Materials and Methods). Readings were taken at the indicated days and log_10_ transformed to meet ANOVA assumptions. Graphs were plotted based on the mean of log_10_-transformed readings for each group at each time point. The corresponding standard errors were very small and not plotted. ANOVA indicated that mean log_10_-transformed readings of the control group were higher than those of both ECD shRNA no. 1 and ECD shRNA no. 2 at days 6, 8, and 10 (all Tukey’s adjusted *P* values less than 0.0001). (D) For colony formation assay, 10,000 cells were plated in 6-well plate, and colonies were fixed and then stained with crystal violet after 10 days of plating. (E and F) Colonies were counted and presented as histograms for BT-474 (E) and SKBR3 (F) cells. ANOVA indicated that the mean number of colonies of the control group was higher than those of both ECD shRNA no. 1 and ECD shRNA no. 2 (all Tukey’s adjusted *P* values less than 0.001). (G and H) Soft agar colony formation assay was used to measure anchorage dependence; 20,000 cells were plated in 0.3% agarose in 6-well plates for 21 days (described in detail in Materials and Methods), and then colonies were stained with 0.05% crystal violet. Colonies were counted and plotted as histograms for BT-474 (G) and SKBR3 (H) cells. ANOVA indicated that the mean number of colonies of control in soft agar was higher than those of both ECD shRNA no. 1 and ECD shRNA no. 2 (all Tukey-adjusted *P* values less than 0.001). *, *P* ≤ 0.05; **, *P* ≤ 0.01; ***, *P* ≤ 0.001; ns, nonsignificant. Means ± SEs were derived from three different experiments, each performed in triplicates.

**FIG 9:**
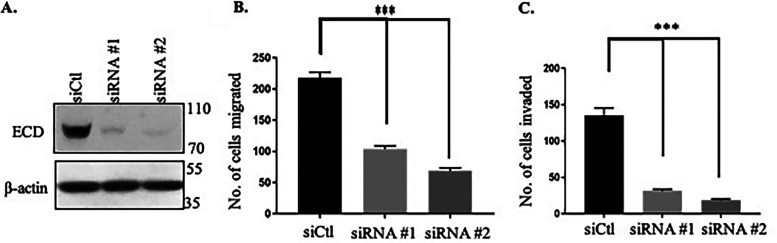
Knockdown of ECD decreases invasion and migration ability of breast cancer cells. BT-474, an ErbB2-positive breast cancer cell line, was treated with control or two independent siRNAs against ECD. (A) Western blotting shows knockdown of ECD with two independent siRNAs. (B and C) Ten thousand cells were counted and plated on Boyden chambers for assessing their ability to migrate (B) and invade (C). After 24 h, cells that had invaded through Matrigel (for invasion assay) and migrated to the bottom surface were fixed and stained with propidium iodide. Pictures were taken, cells were counted, and histograms were plotted. The bar diagrams represent numbers of cells migrated or invaded. ANOVA indicated that the mean number of cells migrated for the control group was higher than those for both ECD siRNA no. 1 and ECD siRNA no. 2 (all Tukey’s adjusted *P* values less than 0.001). *, *P* ≤ 0.05; **, *P* ≤ 0.01; ***, *P* ≤ 0.001; ns, nonsignificant. Means ± SEs were derived from three different experiments, each performed in triplicates.

ECD protein is overexpressed in a significant subset of breast cancers and is also upregulated in other cancers (38–41), and its overexpression correlates with poor prognosis and short survival in ErbB2-overexpressing breast cancers (39). Thus, our findings that ECD is required for the nuclear export of the mRNA for this driver oncogene and to maintain high ErbB2 mRNA and protein levels in ErbB2-overexpressing breast cancer cells suggested the strong likelihood that ECD would be required for ErbB2-driven oncogenic traits. We therefore assessed the functional consequences of ECD KD on key *in vitro* oncogenic traits of ErbB2-overexpressing breast cancer cell lines. ECD KD in BT-474 and SKBR3 cell lines (Fig. 8A) significantly decreased cell proliferation (Fig. 8B and C) (significant at days 6, 8, and 10), colony formation (Fig. 8D, E, and F) (measured after 10 days of seeding), and anchorage-independent growth as measured by the soft agar colony growth assay (Fig. 8G and H) (measured 21 day after seeding). In addition, ECD KD cells exhibited a significant decrease in their ability to migrate across Transwell chambers (Fig. 9B) and to invade through Matrigel (Fig. 9C). Taken together, these results demonstrate that ECD is needed for ErbB2 mRNA export and that optimal expression translates into a requirement of ECD for ErbB2-driven oncogenesis.

## DISCUSSION

In this study, we provide evidence that ecdysoneless (ECD), a protein evolutionarily conserved from yeast to humans, is required for export of mRNAs from the nucleus to the cytoplasm through its interaction with DDX39A. Consequently, ECD KD in ErbB2^+^ breast cancer cells led to decreases in ErbB2 mRNA levels and its stability, which was associated with impairment of oncogenic traits.

First, using mass spectrometry analyses of affinity-purified ECD, we identified a number of ECD-associated proteins known to be involved in mRNA processing, including mRNA export (see Table S1A and B in the supplemental material). The ECD-associated proteins included DDX39A, a known mRNA export regulatory protein. We confirmed the interaction of ECD with DDX39A, using immunoprecipitation followed by Western blotting, immunofluorescence colocalization, and GST fusion protein pulldown experiments. ECD truncation mutational analyses suggested that the middle part of ECD (amino acids 150 to 438) is where ECD interacts with DDX39A (Fig. 1G). However, further detailed mutational analyses will be needed to more precisely localize the region/motif within ECD that interacts with DDX39A. Notably, a single amino acid change (ECD-P617G), modeled after a Drosophila *ecd* mutant previously shown to cause molting defects (50), lacked the ability to interact with DDX39A even though the mutation is not within the DDX39A-interacting region of ECD. Since this mutant retained the ability, like wild-type ECD, to associate with the RUVBL1 component of the R2TP complex (Fig. 1G), it is unlikely that the lack of association with DDX39A is due to misfolding of this protein, although structural studies will be needed to rule this out definitively. It is possible that the conformation adopted by the C-terminal region of ECD impacts the ability of N-terminal sequences to engage in protein-protein interactions. Notably, our previous analyses using SAXS revealed that the first 400 residues of ECD form a globular structure, while the adjacent ∼100 residues were in an extended cylindrical conformation (33), suggesting ECD may function as a scaffolding protein. These structural features suggesting the flexibility of the C-terminal region and the more compact structure in the area where DDX39A interacts are consistent with the potential interactions between N- and C-terminal regions of ECD affecting its interactions, in this case, with DDX39A. Consistent with this possibility, ECD interaction with RUVBL1 involves the more proximal N-terminal sequences (1 to 155) that are dispensable for DDX39A interaction (Fig. 1G).

The multipronged analyses discussed above revealed that ECD exists as a part of protein complexes previously implicated in mRNA export. Multiple approaches supported this prediction: ECD depletion and mRNA export using FISH analyses of poly(A) mRNA (Fig. 3) and biochemical fractionations of nuclear and cytoplasmic mRNA (Fig. 7) showed that ECD is required for mRNA nuclear export. Use of siRNA-mediated ECD KD, inducible ECD KD in human cell lines, and adenovirus-Cre mediated *Ecd* knockout in *Ecd^fl/fl^* MEFs provided complementary approaches to lend strong support for a requirement of ECD in mRNA export. Furthermore, restoration of blocked mRNA export in *Ecd^fl/fl^* MEFs subjected to Cre-mediated *Ecd* deletion by reexpressing exogenous human ECD (Fig. 4) strengthened the conclusion that ECD is critical in mRNA export. Notably, overexpressing exogenous DDX39A in Ecd-deleted MEFs did not restore mRNA export, suggesting that ECD may also interact with other proteins involved in the mRNA export process. Consistent with this possibility, we observed ECD interaction with several RNA processing proteins, including DDX39B (Table S1A and B). However, interaction with DDX39A appears to be important for ECD’s role in mRNA export, as the ECD-P617G mutant, which did not associate with DDX39A (Fig. 1G), was unable to rescue the block in mRNA export in Ecd-deleted MEFs (Fig. 4A and B). Our results are consistent with the impact of ECD-associated protein REF/ALY depletion, which results in mRNAs in the nucleus (64, 65). Given the association of ECD with CRM1 and ALY, future studies are warranted to assess the potential CRM1-dependent versus -independent roles of ECD in mRNA export (24, 66).

Using genetic knockout studies, we have previously shown a physiological role of ECD in embryogenesis, cell cycle regulation, and cell survival (31, 32, 37) and *Drosophila Ecd* is also critical in embryonic development and cell survival (50). To what extent the role of ECD in mRNA export is important in its physiological functions remains conjectural at present and will require further structural and mutational information that might selectively eliminate its mRNA export versus other functions, such as its role in mitigating endoplasmic reticulum stress (32), roles mediated through interactions with the R2TP complex, and others. In the context of mRNA processing itself, which involves integrated events, including transcription, capping, splicing, and transport, and other processing events to allow the availability of translation-ready mRNA in the cytoplasm (4–6, 67), it will be of considerable interest to determine to what extent the new role of ECD in mRNA export we describe here is linked to or distinct from its PRPF8-dependent role in mRNA splicing (30). ECD’s previously established interaction with the PIH1D1 and RUVBL1 components of the R2TP cochaperone complex (37) may allow it to play an even broader role in RNA biogenesis. The R2TP complex is known to promote the assembly of small nucleolar ribonucleoproteins (snoRNPs) (34, 35, 68–73). Notably, ECD was also reported to interact with ZINHIT2 protein, which was shown to function as a mediator of R2TP/prefoldin-like cochaperone interaction with the U5 snRNP (34, 72), a central component of the spliceosome (35). Thus, the ECD-R2TP complex could play a role in promoting mRNA splicing. Our present studies link ECD with multiple mRNA processing proteins involved from splicing to translation of mRNA, and future studies will help decipher a potential role for the ECD-R2TP interaction in mRNA export, splicing, and possibly other aspects of RNA biogenesis.

ECD protein is overexpressed in multiple cancers (38, 39, 41). As heightened metabolic, migratory, proliferative, and other requirements of cancer cells require increased demand on mRNA processing for both driver oncoproteins and general biochemical pathways (74–81), one mechanism by which overexpressed ECD may contribute to oncogenic drive is to ensure efficient mRNA export that is linked to aid and enable oncogenesis (81). In breast cancer, ECD protein is overexpressed and its overexpression is correlated with shorter survival, particularly in ErbB2-overexpressing patients (39). However, it remained unclear if the ECD protein overexpression was related to increase mRNA expression. Using publicly available TCGA and METABRIC data sets, we demonstrate *ECD* mRNA is overexpressed in BC and its overexpression predicted shorter survival in ER^−^, PR^−^, and TNBC as well as HER2^+^ BC subtypes (Fig. 5). Association of higher *ECD* mRNA expression with lymphovascular invasion (LVI), NPI group 3, and lymph node stage 3 (Table S2A) and the ability to independently predict poorer patient outcomes (Table S2B) supported a potentially important role of ECD in BC tumorigenesis. Importantly, our findings that ECD overexpression in BC reflects increased mRNA levels provided a strong rationale to mechanistically examine how ECD regulates ErbB2 mRNA.

As ErbB2 overexpression in most breast cancer patients represents increased transcription from an amplified *ErbB2* gene locus (82), the oncogenic drive is likely to depend on efficient processing of the mRNA for the driver oncogene ErbB2. Our analyses demonstrate that ECD is indeed critical for ErbB2 mRNA export. ECD KD markedly reduced the levels of ErbB2 mRNA but not the levels of EGFR mRNA, even in a cancer cell line with EGFR overexpression (Fig. 6B). FISH analyses confirmed the requirement of ECD for ErbB2 mRNA nuclear export (Fig. 7H and I). Analysis of mRNA levels in cytoplasmic and nuclear fractions further demonstrated the requirement of ECD for ErbB2 mRNA export (Fig. 7A to G). Similar results were observed upon DDX39A KD (Fig. 7A to G). The marked reduction in ErbB2 mRNA levels upon ECD KD reflected destabilization of ErbB2 mRNA secondary to its nuclear retention (Fig. 6E and F), as nuclear export block is known to trigger degradation of certain mRNAs (83). Notably, ECD KD also decreased the levels of exogenously overexpressed ErbB2 (Fig. 6C and D), thus eliminating the possibility of transcriptional regulation of ErbB2 expression by ECD.

Notably, not only full-length ErbB2 but also ECD KD altered the expression levels of two ErbB2 splice variants. One splice variant, Δ16HER2, is known to be highly tumorigenic compared to other isoforms and has been associated with increased invasive and metastatic properties and even trastuzumab resistance (84). ECD KD reduces the level of the Δ16HER2 variant and may also be attributable to the decrease in oncogenic potential of ErbB2^+^ cell lines. However, ECD KD resulted in increased levels of herstatin, which acts as a tumor suppressor by effectively blocking HER2 activity and cell proliferation while promoting apoptosis (58, 59), supporting the notion that ECD is an important player in ErbB2-mediated oncogenesis.

ECD-dependent ErbB2 mRNA export is functionally relevant in the context of overexpressed ECD in cancer cells, which is supported by our observations that KD of ECD dramatically reduced the oncogenic traits (Fig. 8 and 9). These results are consistent with our previous results that ECD provides a co-oncogenic function with mutant Ras protein to fully transform the nontumorigenic immortal human mammary epithelial cells (42). Notably, overexpression of DDX39A was reported to regulate growth and metastasis of various cancer types (46, 49, 76–79). Other studies have shown that DDX39A is also required to maintain the genome integrity and telomere protection, processes that are rendered aberrant as cells become cancerous (85–88).

Taken together, our studies reveal a novel role of ECD, an independent prognostic marker in breast cancer patients, as an essential and important component of the mRNA export machinery. Using ErbB2-overexpressing breast cancer cell lines, we implicate ECD as a regulator of ErbB2 mRNA export and stability to promote ErbB2-driven oncogenic traits. Given the importance of ECD in the cell cycle, cell survival, and embryogenesis and its overexpression across human cancers, our findings provide a new mechanism that could help understand the physiological and pathological roles of ECD.

## MATERIALS AND METHODS

### Biochemical reagents.

Formaldehyde solution (F8775), doxycycline (DOX; D9891), puromycin (P8833), Triton X-100 (T9284), and IGEPAL CA-630 (NP-40; I3021) were from Sigma-Aldrich. Hygromycin B (10687010) was purchased from Thermo Fisher Scientific. Anti-FLAG M2 affinity gel (A2220) and FLAG peptide (3× FLAG; F4799), Met-Asp-Tyr-Lys-Asp-His-Asp-Gly-Asp-Tyr-Lys-Asp-His-Asp-Ile-Asp-Tyr-Lys-Asp-Asp-Asp-Asp-Lys, were from Sigma-Aldrich. Glutathione-Sepharose 4B beads (17-0756-01) and PreScission protease (27-0843-01) were from GE Healthcare.

### Antibodies.

The mouse monoclonal antibody against ECD was previously described (31, 39). The antibodies used in these studies include those against PRPF8 (ab79237), DDX39A (ab96621), LRPPRC (ab97505), and ALY (ab202894) and were from Abcam. Antibodies against CRM1 (46249) and ErbB2 (2242S) were purchased from Cell Signaling. For immunofluorescence staining, we used anti-ECD (catalog number  HPA006465; Sigma-Aldrich) and anti-DDX39 (catalog no. sc-271395; Santa Cruz Biotechnology) antibodies; secondary antibodies tagged with Alexa Fluor 488 (A32731 and A32723) and Alexa Fluor 568 (A-11011 and A-11031) and anti-FLAG antibody (F3165) were from Sigma-Aldrich.

### Cell culture, reagents, and transfections/infections.

Immortal human mammary epithelial cell lines (hMECs) MCF10A and 76NTERT were cultured in DFCI-1 medium (32, 42). HEK-293T cells (ATCC) were maintained in Dulbecco’s modified Eagle’s medium (DMEM) supplemented with 10% fetal bovine serum (48). Adenoviruses encoding enhanced green fluorescent protein-Cre (adeno-GFP-Cre) or enhanced green fluorescent protein (adeno-GFP; control) were purchased from the University of Iowa Gene Transfer Vector Core. For ECD knockout studies, previously published *Ecd^fl/fl^* MEFs were maintained in DMEM supplemented with 10% fetal bovine serum and infected with control or GFP-Cre as described previously (31, 32). For tetracycline (TET)-inducible ECD overexpression, a full-length human ECD cDNA was cloned into the pRev-TRE (Hygro) retroviral vector downstream of the tetracycline response element (TRE). Constructs containing ECD or the empty vector or the pMSCV-rtTA construct encoding the reverse tetracycline transactivator were transfected into the Phoenix Amphotropic (AMPHO) packaging cell line using a standard calcium phosphate transfection protocol to generate retroviral supernatants. The pRev-ECD or pRev control retroviruses were cotransduced with pMSCV-rtTA virus into MCF10A cells using three serial infections with the retroviral supernatants at 12-h intervals followed by selection with 2 μg/ml puromycin and 3 μg/ml hygromycin for 48 h or until all uninfected cells died. To induce the ECD overexpression, vector- or ECD-overexpressing MCF10A cell lines were cultured with 1 μg/ml of doxycycline (DOX), as published previously (32). SKBR3, BT-474, and MDA-MB-468 breast cancer cell lines were purchased from ATCC (Manassas, VA). SKBR3 and BT-474 cells were cultured in RPMI medium supplemented with 10% fetal bovine serum. MDA-MB-468 breast cancer cell line and mouse embryonic fibroblast cells (MEFs) were cultured in DMEM with 10% serum (89). Mycoplasma contamination was examined by 4′,6-diamidino-2-phenylindole (DAPI) staining/fluorescence microscopy. For siRNA-based knockdown, cells were transfected with 30 nM ECD siRNA-1, AAGGCCTAATGAGTCAGATTC-deoxyribosylthymine (dT)dT, ECD siRNA-2, AAGAACCAAGUGGAACCUGUAdTdT, control siRNA (sc-37007), or DDX39A siRNA (sc-77111), which were purchased from Santa Cruz Biotechnology, using the DharmaFECT 1 transfection reagent (T-2001-03; Dharmacon, Pittsburgh, PA).

### Generation of ECD shRNA knockdown cell lines.

ErbB2-overexpressing breast cancer cell lines, SKBR3 and BT-474, were infected with retroviral supernatants corresponding to two distinct ECD shRNAs or a control shRNA, as described previously (38). Virally transduced cells were selected in 0.5 μg/ml puromycin for 3 days, and the expression of endogenous ECD was assessed in whole-cell lysates using Western blotting with an anti-ECD monoclonal antibody (31, 39, 42).

### CellTiter-Glo luminescent cell viability assays.

Two thousand cells of SKBR3 or BT-474 cell lines expressing the control or ECD shRNAs were seeded per well in 96-well plates (Thermo Scientific, catalog no. 136101) and cultured with changes of medium on alternate days. At the designated times, viable cells were quantified using the CellTiter-Glo luminescent cell viability assay according to the manufacturer’s protocol (Promega).

### Colony formation assays.

SKBR3 and BT-474 cell lines expressing the control or two distinct ECD shRNAs were plated (10,000 cells per well) in six-well plates for 9 days with changes of medium on alternate days. The cells were then fixed and stained with 0.25% crystal violet (in 25% methanol) and then imaged, as described previously (89). Pictures were taken and colonies were counted manually.

### Anchorage-independent growth assays.

Twenty thousand cells suspended in RPMI medium containing 0.3% agarose were seeded on top of a bottom layer of 0.6% agarose in 6-well plates, as described previously (89, 90). Each cell line was plated in triplicates, and each experiment was repeated three times. Cultures were fed every 2 days. Twenty-one days after cell seeding, the plates were fixed and stained with 0.05% crystal violet in 25% methanol. Pictures were taken and colonies were counted manually (90).

### Transwell migration and invasion assays.

For migration assays, BT-474 cells transiently transfected with control or ECD-specific siRNAs (described above) were serum-starved in medium with 0.1% FBS for 24 h, and 10,000 cells were plated in top chambers of BD BioCoat Transwell chambers (catalog no. 354578; BD, San Jose, CA). After 2 h, 10% FBS-containing medium was added to the bottom chambers. Migration was then assessed after 24 h by removing the nonmigrated cells on the top surface of the filters, fixation in ice-cold methanol, and staining with propidium iodide, as previously described (42). The filters were mounted on coverslips, and migrated cells on the bottom surface were observed at ×10 magnification under a rhodamine filter using a LSM 710 confocal microscope. Invasion assays were performed as for the migration assay, except for the use of BD Matrigel invasion chambers (no. 354480; BD).

### Immunoprecipitation and immunoblotting.

For immunoprecipitations, cell extracts were prepared in Triton lysis buffer (20 mM Tris-HCl [pH 7.5], 200 mM NaCl, 1% Triton X-100, and a protease inhibitor cocktail from Roche), and protein concentration was determined using a bicinchoninic acid assay (Pierce BCA protein assay kit, 23225). One-milligram aliquots of lysate protein was immunoprecipitated with 5 μg of antibodies overnight at 4°C. The immune complexes were captured with protein A/G-agarose (sc-2003; Santa Cruz Biotechnology) for an additional 2 h. Samples were prepared by boiling the beads in 2× sample buffer and loaded in SDS-PAGE gels, and the gels were transferred to Immobilon transfer membrane (Millipore; IPVHOOO10), which was blotted with corresponding antibodies and developed with enhanced chemiluminescence substrate (PerkinElmer; NEL05001EA).

### GST pulldown assays.

GST fusion proteins of ECD or its mutants were purified from bacterial lysates using glutathione-Sepharose 4B, and GST pulldown experiments were performed as described previously (31, 37).

### RNA isolation and quantitative real-time PCR.

Total RNA was isolated from control and siRNA-treated SKBR3 and BT-474 cell lines using TRIzol reagent (Invitrogen; cat no. 15596018.) according to the manufacturer’s protocol. One microgram RNA was reverse transcribed using SuperScript II reverse transcriptase (Invitrogen, Thermo Fisher Scientific, Waltham, MA) and oligo(dT) primers (18-mer), and aliquots were used for quantitative real-time PCR (qRT-PCR) analyses with specific primer sets (Sigma, St. Louis, MO) listed below. qRT-PCR was carried out in an Applied Biosystems 7500 real-time PCR system using Power SYBR green master mix from Applied Biosystems (Thermo Fisher Scientific). Primers used in this study include the following: *ECD*, ACTTTGAAACACACGAACCTGGCG (forward [F]) and TGATGCAGGTGTGTGCTAGTTCCT (reverse [R]); *DHFR*, TAAACTGCATCGTCGCTGTGT (F) and AGGTTGTGGTCATTCTCTGGAAA (R); *ErbB2*, AGCCTTGCCCCATCAACTG (F) and AATGCCAACCACCGCAGA (R); *EGFR*, TGCCATCCAAACTGCACCTA (F) and CTGTGTTGAGGGCAATGAG (R); *GAPDH*, GTCATCCATGACAAGTTTGG (F) and TGCCAGTGAGCTTCCCGTTC (R); 18S rRNA, GCTTAATTTGACTCAACACGGGA (F) and AGCTATCAATCTGTCAATCCTGTC (R); *RNU1* snRNA, ATACCATGATCACGAAGGTGGTT (F) and CAGTCCCCCACTACCACAAATTA (R); *HER2*-Δ16, CACCCACTCCCCTCTGAC (F) and GCTCCACCAGCTCCGTTTCCTG (R); and herstatin, AGCTGTGTGACTGCCTGTCCCT (F) and GTACCCACTCACTGCCCCCGAGG (R).

### RNA fluorescence *in situ* hybridization analysis.

Cells seeded on coverslips were transfected with control or siRNAs against ECD and DDX39A, cultured for 72 h, fixed with RNase-free 3.7% formaldehyde, and permeabilized in 70% RNase-free ethanol overnight at 4°C. The coverslips were prehybridized for 5 min (with wash A buffer from the Stellaris, LGC Biosearch Technologies kit) and hybridized with 12.5 μM oligo(dT) Quasar 570 [22-nucleotide [nt] oligo(dT) custom probe in hybridization buffer; Stellaris, LGC Biosearch Technologies] for 4 h at 37°C in dark. The coverslips were incubated with wash A buffer for 30 min at 37°C in the dark and wash B buffer at room temperature for 5 min, according to the manufacturer’s instructions. The coverslips were mounted with Vectashield-DAPI mounting medium and imaged using the LSM 710 confocal microscope under a 63× lens. For ErbB2 FISH, we used probe (no. VSMF-2102-5) from Stellaris, LGC Biosearch Technologies. Quantification of nuclear versus cytoplasmic fluorescence was performed using the Intensity Ratio Nuclei Cytoplasm plugin in Image J software.

### RNA localization by nuclear/cytoplasmic fractionation of cells.

After the specified treatments, cells were trypsinized, counted, and divided into two equal aliquots (one for total RNA isolation and the second for nuclear and cytoplasmic fractionation). Both cell aliquots were lysed on ice in cell lysis buffer for 10 to 20 min. For nuclear/cytoplasmic fractionation, the lysate was centrifuged at 14,000 rpm to collect the nuclear pellet and soluble fraction. The fractions as well as the lysate for total RNA isolation were treated with buffer G (RNA isolation kit cat no. 25501; Active Motif) in 70% ethanol, loaded on to a spin column, and subjected to centrifugation; the retained precipitate was washed with ethanol one time and was eluted in RNase-free water. cDNAs were prepared from various RNA preparations, and qRT-PCR was performed (as mentioned above). mRNAs from nuclear and cytoplasmic fractions were normalized to total RNA isolated from an identical number of cells.

### mRNA stability assays.

Cells were seeded in 6-well plates (200,000 cells per well). After 48 h of ECD knockdown, actinomycin D (5 μg/ml) (Sigma, St. Louis, MO) was added at zero time point. Total RNA was isolated at indicated time points (0, 2, 4, 6, 8, and 12 h) after actinomycin D treatment with TRIzol per the manufacturer’s protocol. First-strand cDNA was synthesized using SuperScript III reverse transcriptase, followed by qRT-PCR with ErbB2, ECD, and GAPDH primers, as described above.

### TCGA and METABRIC database analysis.

The *ECD* mRNA expression analysis in breast cancer tissues was performed using the publicly available fragments per kilobase of transcript per million mapped reads upper quartile (FPKM-UQ) data from The Cancer Genome Atlas (TCGA), downloaded from the Genomic Data Commons (GDC). For the analysis of ECD mRNA expression, the BC TCGA data set comprised 1,089 cases, which were further stratified as 676 cases of ER^+^/PR^+^, 164 cases of HER2^+^, and 112 adjacent normal breast tissue samples based on clinical data. ECD expression in box plots represents the log base 2 (log_2_) values compared to that for adjacent normal tissue, and the significance was calculated using a *t* test. The survival data by multivariate Cox regression analysis of the 854-case TCGA cohort were analyzed as described previously (91). The number of cases analyzed per subgroup is indicated in the Kaplan-Meier plots. Dichotomization of *ECD* expression was performed using the X-tile software to compare the outcomes (92). For the TCGA data set, 834.2 was used as a cutoff point for high and low *ECD* mRNA expression. The *ECD* mRNA expression was also assessed utilizing the Molecular Taxonomy of Breast Cancer International Consortium (METABRIC) cohort (*n* = 1,980) with the clinical-pathological parameters and outcome data available (93). The high and low *ECD* expression was assigned a cutoff of 7.24 using the X-tile software.

### Statistical analysis.

Each assay in our study was repeated at least three independent times. Comparisons between two groups were made using *t* tests for continuous outcomes. Comparisons among at least three treatment groups were made using one-way analysis of variance (ANOVA) for continuous outcomes. If the overall tests yielded significant results, *post hoc* tests with Tukey’s method for multiple comparisons were conducted. Log_10_ transformations were applied to continuous outcomes to meet ANOVA or *t* test assumptions as necessary. *P* values of ≤0.05 were considered statistically significant. Data analysis was performed using SAS version 9.4 (SAS Institute, Cary, NC, USA) and GraphPad Prism. SPSS 24.0 statistical software (SPSS Inc., Chicago, IL, USA) was used for statistical analysis of *ECD* mRNA expression. Survival curves were analyzed using the Kaplan-Meier analysis with the log rank test. Cox’s proportional hazard method was performed for multivariate analysis to identify the independent prognostic value of *ECD* expression. Spearman correlation coefficients were calculated to analyze the association between continuous variables. The chi-square test was carried out for interrelationships between categorical variables. A *P* value of <0.05 was considered statistically significant.
