# Supplementary material for: The Mammalian Ecdysoneless Protein Interacts with RNA Helicase DDX39A To Regulate Nuclear mRNA Export
Source: Mol Cell Biol. 2021 Jun 23;41(7):e00103-21. doi: 10.1128/MCB.00103-21 (PMC8224239; doi:10.1128/MCB.00103-21)
Supplement: Supplemental file 1 — Tables S1 and S2. Download MCB00103-21_Supp_1_seq13.pdf, PDF file, 0.7 MB [file mcb00103-21_supp_1_seq13.pdf]

Supplemental Material:

Table 1A. List of ECD interacting proteins identified by *in vitro* tandem affinity purification followed by mass spectrometry (Highlighted are the proteins involved in RNA processing)

GenInfo Identifier	Identified protein	MW (Da)	Mascot score
gi37492	Alpha-tubulin	50126	6711
gil22507514	Eukaryotic translation elongation factor 1 alpha 1	50109	4758
gi113654237	Protein kinase, DNA-activated, catalytic polypeptide	468788	4193
gi4507729	Tubulin, beta 2	49875	3918
gi162896589	Eukaryotic translation elongation factor 1 alpha 1 variant	50093	2018
gi14210536	Tubulin, beta 6	49825	1565
gil30046984	MYB binding protein 1a (MYBBP1A)	148781	1505
gi30582781	Tubulin, beta 4	88324	1330
gi150345984	ATP synthase, H+ transporting, mitochondrial F1 complex, alpha subunit 1	59714	1317
gi113543589	Tubulin, alpha 6	49863	966
**gi31621305**	**Leucine-rich PPR motif-containing protein (LRPPRC)**	**157805**	**883**
gil24308324	Fatty acyl CoA reductase 1 (FAR1)	59319	721
gi296908	Replication factor C 1 (RFC1, PO-GA)	128203	621
gi132189394	ATP synthase, H+ transporting, mitochondrial F1 complex, beta subunit precursor	56525	598
gi23398599	Patatin-like phospholipase domain containing 6 (PNPLA6)	146727	556
gi154696624	Eukaryotic translation initiation factor 4A, isoform 1	46125	462
gi289581 18	Structural maintenance of chromosomes 3 (SMC3)	141420	409

gi999380	Structural maintenance of chromosomes 1A (SMC1A)	1431 16	404
gi4758668	Serine palmitoyltransferase, long chain base subunit 2 (SPTLC2)	62884	390
**gi11905998**	**Nuclear RNA helicase (DDX39A)**	**49046**	**282**
gi14240263	Fas associated factor family member 2 (FAF2)	52388	279
gi37926025	Vacuola r protein sorting 4 homolog B (VPS4B)	49271	278
gi15454084	Serine palmitoyltransferase subunit 1 isoform a (SPTLC1)	52710	262
gil50345982	ATP synthase, H+ transporting, mitochondrial F1 complex, alpha subunit isoform b	54460	238
gil4557303	Aldehyde dehydrogenase 3A2 isoform 2 (ALDH3A2)	54813	213
gi76879704	24-dehydrocholestero l reductase (DHCR24, Seladin1)	49404	186
gi1297274	Tubulin, beta 3	50485	186
gi113325168	Beta-1,4-mannosyltransferase (ALG1)	52511	185
gi115277588	TATA binding protein interacting protein 49 kDa (TIP49)	50196	168
gil16877130	Sulfide dehydrogenase like (SQR DL)	49929	153
gi119924129	RAD50 homolog isoform 1 (RAD50)	153797	147
gil1265327 1	Thyroid hormone receptor interactor 13 (TR IP13)	48520	129
gi121327708	Nucleosome assembly protein 1-like 1 (NAP1L1)	45346	125
gi56206959	Tubulin, alpha-like 3	49877	124
gil52222822	Lysophosphatidylcholine acyltransferase 4 (LPCAT4)	57183	120
gi27363457	AarF domain containing kinase 4 (ADCK4)	60031	119
gi18922701	Acylglycerol kinase (AGK)	47107	116
gi110433931	Lyso phosphatidylcholine acyltransferase 1 (LPCAT1)	26025	115

gi10435296	Atlastin GTPase 3 (ATL3)	60491	110
gil30749718	Glutamate Dehydrogenase 1 (GLUD1)	54916	99
gi17511683	ATPase family, AAA domain containing 38 (ATAD3B)	72555	93
gi55958454	Structural maintenance of chromosomes 2 (SMC2)	135572	92
gi39645762	Solute carrier family 25, member 4 (SLC25A4, ANT1)	33043	73
gi15381417	Vacuolar protein sorting 4 (VPS4)	48318	68
**gi160594465**	**Chain A, Structure of Deed to Dead Mutation of Human Uap56 (BAT1, DDX39 B)**	**44859**	**67**
gi62089230	Solute carrier family 2, member 6 (ANT3)	35361	65
gi115488941	Glial fibrillary acidic protein (GFAP)	49850	65
gil37589312	AarF domain containing kinase 1 (ADCK1)	59681	63

Table IB. List of ECD interacting proteins identified by <u>in vivo immunoprecipitation</u> followed by mass spectrometry (Highlighted are the proteins involved in RNA processing)

NCBI accession number	Identified protein	MW	Peptide matches
**NP-054733**	**U5 small nuclear ribonucleoprotein 200, kDa helicase (ASCC3L 1, BRR2)**	**244495 .8**	**33**
**NP-006436**	**PRP8 pre-mRNA processing factor 8 1homolog, S. cerevisiae (PRPF8)**	**273590 .6**	**23**
**AK09067 1**	**SPLICING HELICASE BRR2**	**71456.5**	**16**
**NP-1136077**	**116 kDa U5 small nuclear ribonucleoprotein component (EFTUD2, Snu114)**	**109420. 1**	**15**
NP-005667	RNA binding motif protein 10 (BRM10)	110322	13
NP-002218	Janus kinase 1 (JAK1)	133421.7	10
NP-004492	heterogeneous nuclear ribonucleoprotein U isoform b	88962 .3	5
NP-001120680	tet oncogene family member 2 (TET2)	130234 .1	4
NP-057460	ankyrin repeat and FYVE domain containing 1 isoform 1 (ANKFY 1)	128470 .7	4

Table 2A: Associations between *ECD* mRNA expression and clinicopathological variables from METABRIC & TCGA breast cancer dataset

Clinicopathological Criteria	METABRIC cohort			TCGA cohort		
	ECD mRNA expression N (%)		χ2 (p Value) Significant p values (shown in red)	ECD mRNA expression N (%)		χ2 (p Value) Significant p values (shown in red)
	Negative/ Low Expression	High Expression		Negative/ Low Expression	High Expression	
**Age at Diagnosis**						
< 50 years	377 (88.9)	47 (11.1)	0.000	171 (74.0)	60 (26.0)	0.566
≥ 50 years	1383 (88.9)	173 (11.1)	(0.985)	445 (71.4)	178 (28.6)	(0.452)
**Tumour Size (cm)**						
< 2.0	555 (89.2)	67 (10.8)	0.113	103 (68.2)	48 (31.8)	1.814
≥ 2.0	1187 (88.7)	151 (11.3)	(0.736)	339 (73.9)	120 (26.1)	(0.178)
**Nodal Stage**						
Stage 1	889 (90.1)	98 (9.9)	4.555	114 (70.4)	48 (29.6)	0.453
Stage 2	552 (88.7)	70 (11.3)	(0.103)	364 (72.9)	135 (27.1)	(0.797)
Stage 3	271 (85.8)	45 (14.2)		138 (71.5)	55 (28.5)	
**Histological Grade**						
Grade 1	148 (87.1)	22 (12.9)	3.943	47 (59.5)	32 (40.5)	3.192
Grade 2	697 (90.5)	73 (9.5)	(0.139)	95 (65.5)	50 (34.5)	(0.203)
Grade 3	835 (87.7)	117 (12.3)		110 (71.0)	45 (29.0)	
**Lymphovascular invasion**						
Negative	828 (89.0)	102 (11.0)	0.500	421 (75.3)	138 (24.7)	8.150
Positive	558 (87.9)	77 (12.1)	(0.480)	195 (66.1)	100 (33.9)	(**0.004**)
**Nottingham Prognostic Index groups**						
Good	612 (90.0)	68 (10.0)	6.985			
Moderate	982 (89.2)	119 (10.8)	(**0.030**)			
Poor	166 (83.4)	33 (16.6)				
**PAM50 subtype**						
Luminal A	632 (88.0)	86 (12.0)	16.647	289 (71.4)	116 (28.6)	40.567
Luminal B	417 (85.5)	71 (14.5)	(**0.002**)	76 (53.9)	65 (46.1)	(**1.1476E-7**)
Her2 enriched	217 (90.4)	23 (9.6)		41 (73.2)	15 (26.8)	
Basal Like	299 (90.06)	31 (9.4)		107 (80.5)	26 (19.5)	
Normal like	190 (95.5)	9 (4.5)		25 (83.3)	5 (16.7)	
Unclassified	0 (0)	0 (0)		78 (87.6)	11 (12.4)	
**IntClustMemb**						
IntClustMemb 1	119 (85.6)	20 (14.4)	25.977			
IntClustMemb 2	65 (90.3)	7 (9.7)	(**0.002**)			
IntClustMemb 3	262 (90.3)	28 (9.7)				
IntClustMemb 4	325 (94.8)	18 (5.2)				
IntClustMemb 5	161 (84.7)	29 (15.3)				
IntClustMemb 6	72 (84.7)	13 (15.3)				
IntClustMemb 7	176 (92.6)	14 (7.4)				
IntClustMemb 8	260 (87.0)	39 (13.0)				
IntClustMemb 9	126 (86.3)	20 (13.7)				
IntClustMemb 10	194 (85.8)	32 (14.2)				
**Estrogen Receptor (ER)**						
Negative	426 (89.9)	48 (10.1)	0.612	149 (80.5)	36 (19.5)	8.035
Positive	1334 (88.6)	172 (11.4)	(0.434)	447 (70.0)	192 (30.0)	(**0.005**)
**Progesterone Receptor (PR)**						
Negative	839 (89.3)	101 (10.7)	0.243	209 (76.8)	63 (23.2)	3.974
Positive	921 (88.6)	119 (11.4)	(0.622)	387 (70.2)	164 (29.8)	(**0.046**)
**Human epidermal growth factor receptor 2 (HER2)**						
Negative	1543 (89.0)	190 (11.0)	0.306	412 (70.4)	173 (29.6)	3.558
Positive	217 (87.9)	30 (12.1)	(0.580)	106 (78.5)	29 (21.5)	(**0.059**)
**Triple negative status (TNBC)**						
Non TNBC	1470 (88.6)	190 (11.4)	1.165	499 (70.7)	207 (29.3)	6.938
TNBC	290 (90.6)	30 (9.4)	(0.280)	98 (82.4)	21 (17.6)	(**0.008**)
**P53 mutation**						
Wild type	65 (90.3)	7 (9.7)	5.020			
Mutated	18 (72.0)	7 (28)	(**0.025**)			

Table 2B: Multivariate Cox Regression analysis from all breast cancer specimen within TCGA & METABRIC dataset shows ECD is an independent poor prognostic marker

Covariates	TCGA breast cancer cohort				METABRIC breast cancer cohort			
	P-value Significant p values (shown in red)	Hazard Ratio	95% CI		P-value Significant p values (shown in red)	Hazard Ratio	95% CI	
			Lower	Upper			Lower	Upper
Age	0.133	2.156	0.791	5.878	0.524	0.933	0.754	1.155
Tumour Size.	0.138	2.070	0.791	5.412	**9.7906E -9**	1.899	1.525	2.365
Tumour Grade	0.841	1.076	0.527	2.196	**0.0007**	1.338	1.131	1.583
ER expression	0.083	0.293	0.073	1.173	0.122	0.824	0.644	1.054
PR expression	0.314	1.992	0.521	7.612	**0.004**	0.727	0.583	0.907
HER2 expression	**0.037**	2.678	1.062	6.752	**0.0001**	1.571	1.241	1.989
ECD expression	**0.032**	2.448	1.078	5.560	**0.0435**	1.329	1.008	1.751
