# Supplementary material for: The Mammalian Ecdysoneless Protein Interacts with RNA Helicase DDX39A To Regulate Nuclear mRNA Export
Source: Mol Cell Biol. 2021 Jun 23;41(7):e00103-21. doi: 10.1128/MCB.00103-21 (PMC8224239; doi:10.1128/MCB.00103-21)
Supplement: Supplemental file 2 — Fig. S1. Download MCB00103-21_Supp_2_seq14.pdf, PDF file, 1.5 MB [file mcb00103-21_supp_2_seq14.pdf]

Supporting information

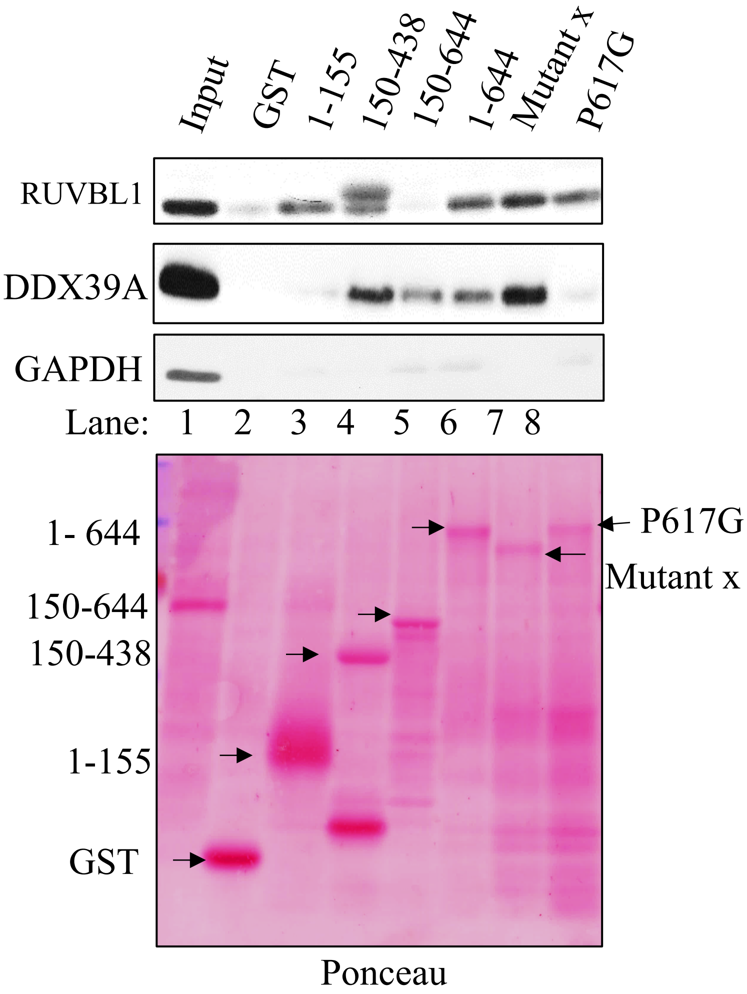

Fig. S1A. Interaction of ECD full length and its mutants with DDX39A. GST or GST fusion with full length ECD or various ECD mutants were incubated with protein lysates of HEK-293T cells transiently transfected with FLAG-DDX39A and FLAG-RUVBL1, following that bound proteins were analyzed by western blotting with the indicated antibodies. The membrane was stained with Ponceau S to visualize the GST fusion proteins (indicated by arrows). The experiment shown is a representative of at least three repeats with comparable results.
